# The Key Role of Chalcogenurane Intermediates in the Reduction Mechanism of Sulfoxides and Selenoxides by Thiols Explored In Silico

**DOI:** 10.3390/ijms24097754

**Published:** 2023-04-24

**Authors:** Andrea Madabeni, Laura Orian

**Affiliations:** Dipartimento di Scienze Chimiche, Università degli Studi di Padova, Via Marzolo 1, 35131 Padova, Italy; andrea.madabeni@studenti.unipd.it

**Keywords:** activation strain model, DFT calculations, disulfide, methionine sulfoxide reductase, selenoxide, sulfoxide, thiol

## Abstract

Sulfoxides and selenoxides oxidize thiols to disulfides while being reduced back to sulfides and selenides. While the reduction mechanism of sulfoxides to sulfides has been thoroughly explored experimentally as well as computationally, less attention has been devoted to the heavier selenoxides. In this work, we explore the reductive mechanism of dimethyl selenoxide, as an archetypal selenoxide and, for the sake of comparison, the reductive mechanism of dimethyl sulfoxide to gain insight into the role of the chalcogen on the reaction substrate. Particular attention is devoted to the key role of sulfurane and selenurane intermediates. Moreover, the capacity of these system to oxidize selenols rather than thiols, leading to the formation of selenyl sulfide bridges, is explored in silico. Notably, this analysis provides molecular insight into the role of selenocysteine in methionine sulfoxide reductase selenoenzyme. The activation strain model of chemical reactivity is employed in the studied reactions as an intuitive tool to bridge the computationally predicted effect of the chalcogen on the chalcogenoxide as well as on the chalcogenol.

## 1. Introduction

**Scheme 1:**
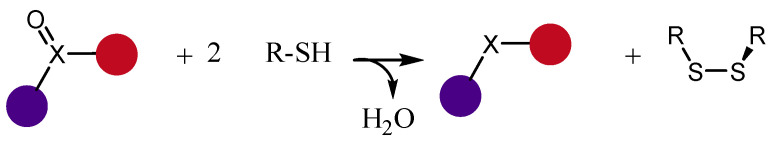
General reaction for the reduction of a chalcogenoxide (X = S, Se) to a chalcogenide, with concomitant oxidation of two equivalents of thiol to disulfide.

Since the 60s, sulfoxides are well known for their capacity of acting as mild oxidizing agents [1]. Selenoxides can engage in a similar, but faster, redox chemistry, and can easily oxidize thiols to disulfides at room temperature [2,3,4,5] (Scheme 1).

Attempts to exploit this reactivity in a glutathione peroxidase (GPx)-like catalytic cycle [6,7] have been pursued and, in these cases, the oxidation of the selenide to selenoxide is usually rate determining, while the reduction from selenoxide to selenide is, as stated above, fast [8,9]. However, selenoxides are more likely to act as pro-oxidants species rather than as intermediates in GPx-like cycles [10]. In particular, Iwaoka and co-workers showed that soluble selenoxides can be used to induce fast and complete disulfide formation in proteins having multiple free reduced thiols [4,9].

In addition to the synthetic value of the reaction, sulfoxides and selenoxides can be produced in vivo by the oxidation of critical methionine (Met) and selenomethionine (SeMet) residues to methionine sulfoxide (MetO) and selenomethionine selenoxide (SeMetO), respectively. These oxidized amino acids are then reduced back either by free thiols or by enzymatic processes. In particular, the reduction of SeMetO has been repeatedly shown to proceed in a fast and spontaneous way at room temperature in the presence of low molecular mass thiols such as glutathione [2,3]. Conversely, MetO requires its own reducing enzyme, i.e., methionine sulfoxide reductase (Msr) [11].

**Figure 1:**
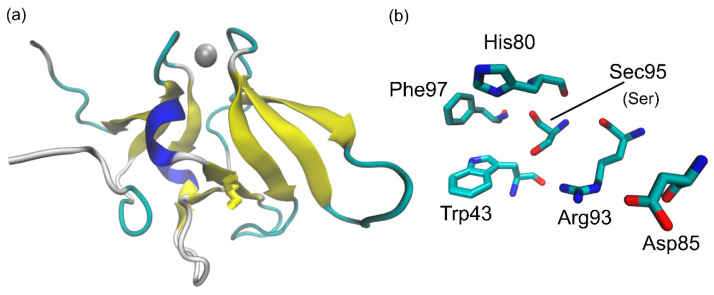
(**a**) Crystal structure of human MsrB1 (PDB: #3MAO) with Sec95 (mutated to Ser95) represented in licorice; and (**b**) close-up on the residues surrounding Sec95, which are known to play a role in Msr catalysis [12].

Msrs are antioxidant repair enzymes which catalyze the reaction shown in Scheme 1. Two structurally different classes of enzymes constitute the Msrs family, MsrA and MsrB, specific for the S and R epimers of MetO, respectively, which are generated by the oxidation of the prochiral sulfur nucleus. While MsrAs display some variability, with enzymes working via one, two, or even three reactive Cys residues, MsrBs usually employ one or two cysteines (Cys) to reduce MetO to Met, namely a catalytic Cys (CysA) and a recycling Cys (CysB). Interestingly, some members of both classes naturally evolved to employ one catalytic selenocysteine (Sec) instead of CysA [12]. In mammals, this is the case of one of the three known MsrB enzymes, i.e., MsrB1, in which Sec95 (numeration refers to Mus Musculus MsrB1) acts as the catalytic CysA (Figure 1). As verified for other selenoenzymes [13] (e.g., GPx4) [14], site-directed mutagenesis proved that the presence of Sec instead of Cys in the active site of human MsrBs provides a kinetic advantage in MetO reduction [12,15].

**Scheme 2:**
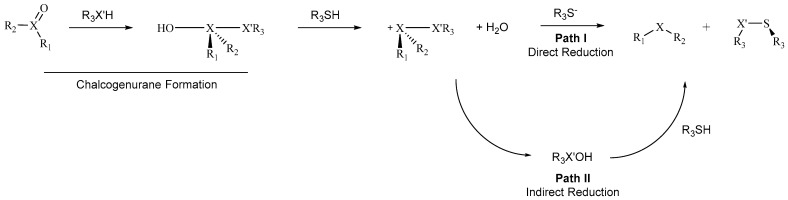
Schematic mechanism for the reduction of chalcogenoxides to chalcogenides. Chalcogenurane (X = S, Se) formation in the first step of sulfoxides and selenoxides reaction with thiols and further direct (path I) or indirect (path II) reduction of the chalconium intermediate to sulfide and disulfide, or to sulfenic acid and sulfide, respectively. In our study, R_1_, R_2_, R_3_ = CH_3_. The enzymatic pathways (which proceed through the same intermediates) can be found in the supporting information (Scheme S1).

Despite possessing diverse structural features, MsrA and MsrB are postulated to reduce MetO along similar reaction pathways, even if different residues might be involved in the stabilization of the transition states and intermediates [12,16,17,18]. First, CysA attacks the sulfur atom of the sulfoxide, while one proton is dislocated from a catalytic pocket residue to the oxygen atom of the sulfoxide moiety. This leads to the formation of a thiosulfurane [19]. This first step is analogous to that postulated for the reduction of sulfoxides by free thiols. Similarly, in the reduction of selenoxides, such as SeMetO, the formation of a selenurane is invoked [3] (Scheme 2).

Then, the chalcogenurane undergoes O–X bond breaking, with the formation of a chalconium cation [16,20]. In MsrA, a suitably activated water molecule was found to act as a nucleophile, converting the catalytic CysA (Sec95) to its sulfenic (selenenic) acid form and leading to the reduced methionine [16]. The sulfenic/selenenic acid is then reduced by condensation with a resolving Cys to a disulfide/selenyl sulfide bond, which is ultimately further reduced by the thioredoxin system to release the ground state Msr enzyme [12]. The formation of a sulfenic acid (RX’OH in Scheme 2) was assessed via X-ray crystallography, mass spectrometry and by trapping experiments [16,21]. Conversely, to the best of our knowledge, no evidence has been so far reported on the formation of this intermediate when the reaction occurs with free thiols, suggesting a direct reduction in which the nucleophilic thiolate attacks the sulfurane/sulfonium cation. However, since sulfenic acids can undergo rapid condensation to disulfides, their involvement in the reaction cannot be completely ruled out [20]. Interestingly, previous computational investigation of the Msr catalytic cycle showed that the formation of sulfenic acid (Path II, Scheme 2) is kinetically disfavored with respect to the direct reduction of the chalconium cation (Path I, Scheme 2), even if both processes have a biologically accessible activation energy [19] and might occur in parallel. In fact, direct reduction was also postulated in the biological literature before the experimental detection of sulfenic acid was reported [11]. The similarities between the reduction mechanism of sulfoxides by free thiols and the catalytic mechanism of Msrs should not come as unexpected, since the plausible mechanism for the enzyme was partly built upon previous small-molecules mechanistic knowledge [11,22].

In this work, we employ density functional theory (DFT) calculations to investigate the mechanism of the reduction of selenoxides, revisiting in silico as well as some known aspects of the reduction mechanism of sulfoxides. Simple molecular models are employed to draw general conclusions about the title reaction and gain insight on the role of the chalcogen. In fact, intrigued by the natural occurrence of Sec in selenoprotein MsrB1, one of the few human selenoproteins with a known function, we also aim to explore the role of Se when a selenol acts as a reducing agent instead of a thiol. Particular attention will be given to the preliminary sulfurane and selenurane formation, which is a key reactive step shared by both the enzymatic and the molecular mechanism. While our small model clearly cannot capture all of the structural complexity of Msr, the intrinsic trends for the effect of the chalcogen can also be expected to be qualitatively transferable in an enzymatic context, as reported for GPx and proteins with peroxidatic cysteines/selenocysteines [23,24,25]. For the other steps of the reduction mechanism (i.e., following the chalcogenurane formation) a more cautious comparison with the enzyme will be undertaken, since the molecular and the enzymatic pathways might at least partially diverge.

## 2. Results and Discussion

To investigate the title reaction, a minimal model was employed consisting of the reduction of dimethyl sulfoxide (DMSO) and dimethyl selenoxide (DMSeO) by two methyl thiols (CH_3_SH). Moreover, to probe the role of Sec95 in MsrB1, the same reaction was investigated with one methyl selenol (CH_3_SeH) and one CH_3_SH. The reaction with two CH_3_SeHs was not investigated, since, to the best of our knowledge, no Msr selenoprotein possesses two active Sec residues. Moreover, the selenol was considered to react first with the chalcogenoxide, to form the chalcogenurane in Scheme 2.

### 2.1. Mechanistic Details

**Scheme 3:**
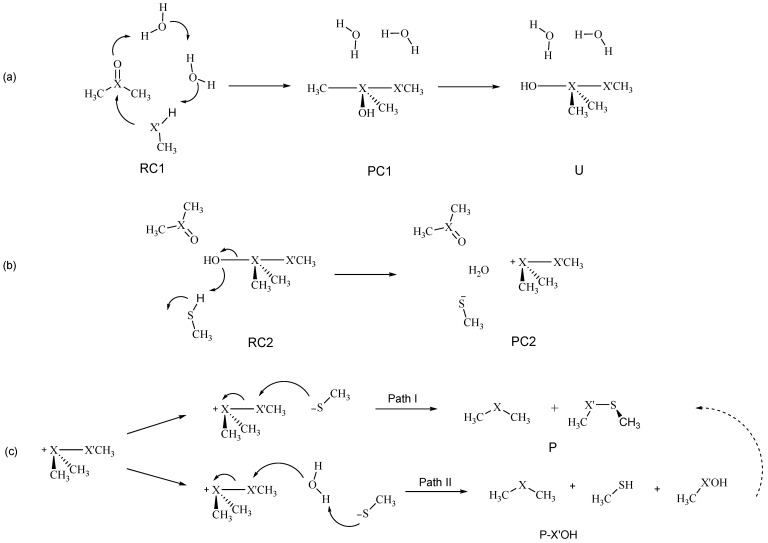
All reaction mechanisms investigated in this work. (**a**) Chalcogenurane (U) formation. (**b**) Chalcogenurane disruption and chalconium cation formation (PC2). (**c**) Chalcogenide (P) release either via a direct mechanism (path I) or indirect mechanism (path II) passing through a sulfenic or selenenic acid (P-X’OH). The condensation of sulfenic acid (dashed arrow) to the final products P was not investigated.

As modeled by us, the mechanism can be described as three progressive steps, i.e., (a) chalcogenurane formation; (b) chalconium cation formation (or chalcogenurane disruption); and lastly (c) sulfide or selenide release (Scheme 3). This pathway closely resembles that investigated by Balta et al. which was limited to DMSO [20]. In a different way, we employed two water molecules to mediate the proton transfer from CH_3_X’H to the chalcogenoxide oxygen as previously done by Bayse for the reduction of seleninic acid [26].

**Figure 2:**
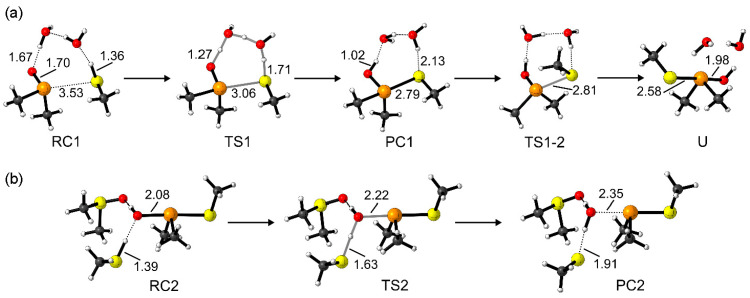
Representative structures along the reduction mechanism of DMSeO by CH_3_SH. (**a**) Selenurane (U) formation; (**b**) selenurane (U) disruption and selenonium cation (PC2) formation. Interatomic distances (in Å) that undergo relevant changes along the reaction are indicated. Level of theory: ZORA-BLYP-D3(BJ)/TZ2P.

Representative structures along the mechanism in the gas phase are displayed in Figure 2. Starting from a reactant complex (RC1), the first step passes through a TS in which the two chalcogens X and X’ come close, and in a concerted fashion the proton of CH_3_X’H is shuttled through the two water molecules to the chalcogenoxide moiety (TS1). A product complex (PC1) is formed, in which the central chalcogen is surrounded by an OH group, the chalcogenolate CH_3_X’, and the two methyl groups. A subsequent isomerization (TS1-2) leads to the canonical chalcogenurane U with a linear O–X–X’ bond.

The O–X bond breaking is modeled starting from an adduct between the second CH_3_SH and U (RC2). The O–X bond of U is then cleaved by a proton transfer from CH_3_SH (TS2), leading to an adduct between methyl thiolate, water, and the chalconium cation (PC2). To stabilize the released water molecule, a second DMSO was employed in RC2, TS2, and PC2, as previously performed by Balta et al. [20] While this choice may seem rather arbitrary, we expect the bulk solvent to take on the same role when the reaction occurs in solution; similarly, specific hydrogen bond acceptor residues can act as DMSOs when the reaction occurs in a protein environment [19]. As shown in Scheme 3, the chalconium cation can then be reduced to chalcogenide either by a direct or indirect pathway. In the gas phase, both reactions proceed without any appreciable activation energy at our level of theory. This can be expected, since the reaction involves two charged reactants–thus, the charge recombination occurs spontaneously, leading either to sulfenic/selenenic or directly to the dichalcogenide and to the sulfide/selenide. Further details about this step will be discussed later on in Section 2.4.

### 2.2. Energetics and Role of the Chalcogen

**Figure 3:**
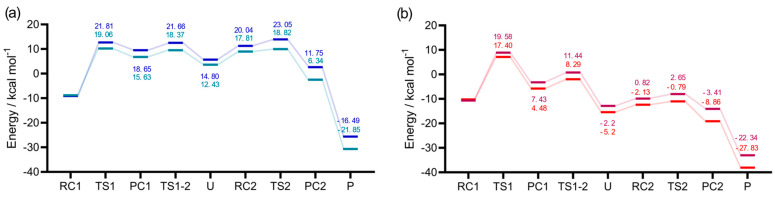
Reaction profiles (kcal mol^−1^). (**a**) Comparison between the reduction of DMSO by CH_3_SH (blue) and CH_3_SeH (turquoise). (**b**) Comparison between the reduction of DMSeO by CH_3_SH (dark red) and CH_3_SeH (orange). Activation and reaction energies with respect to RC1 are shown on the profile. Level of theory: COSMO-M06//BLYP-D3(BJ).

The reaction profiles for the steps described in Section 2.1 were computed for X, X’ = S, Se, in order to provide a comprehensive insight into the reduction of sulfoxides, selenoxides, and into the role of Sec vs. Cys. (Figure 3) First, the DMSO reduction will be compared to the DMSeO reaction when CH_3_SH is the only reducing agent, (Figure 3a, blue vs. Figure 3b, red); then, the features of the potential energy surface (PES) when CH_3_SeH is involved will be discussed (Figure 3a turquoise and Figure 3b orange).

The first step of the reaction is characterized by the highest activation energy of the whole mechanism, i.e., 21.81 kcal mol^−1^ for DMSO + CH_3_SH, and 19.58 for DMSeO + CH_3_SH. Interestingly, after the proton is transferred from the thiol to the chalcogenoxide, DMSeO forms a much more stable adduct with the thiolate than DMSO (PC1). Then, a low energy isomerization transition state connects PC1 to the canonical chalcogenurane with a linear O–X–S arrangement. The difference in stability between DMSO and DMSeO PC1 is mirrored by the stability of U, which is ca. 18 kcal mol^−1^ more stable for DMSeO (−12.88 kcal mol^−1^ with respect to the free reactants, and almost iso-energetic to the corresponding RC1) than for DMSO (lying +5.67 kcal mol^−1^ with respect to the free reactants and almost 15 kcal mol^−1^ higher than the corresponding RC1).

The difference in stability of the two adducts/chalcogenuranes is not surprising, since Se forms more easily hypervalent species than S [13,27], and this appears to be the most striking difference between the two mechanistic profiles.

Considering the chalcogenurane disruption through TS2, DMSO and DMSeO display a very similar activation energy with respect to the closest adduct RC2 (2.65 kcal mol^−1^ vs. 1.83 kcal mol^−1^, respectively). However, TS2 is located way higher on the PES for DMSO than for DMSeO; since it lies even higher than TS1, i.e., 23.05 kcal mol^−1^ above RC1. For DMSO, the chalcogenurane formation and disruption proceed with an overall barrier of ca. 23 kcal mol^−1^. Conversely, for DMSeO, U formation and disruption proceed with an overall barrier corresponding only to the formation of U, i.e., 19.58 kcal mol^−1^. In both cases, however, the chalcogenurane formation appears to be a key step, contributing mostly or totally to the activation energy required for the chalcogenoxide reduction and giving the PES a peculiar shape.

In a somewhat unexpected contrast with these data, kinetic analysis for the reduction of SeMetO showed that U disruption might be slower than U formation [3]. However, SeMetO is present in the solution partly as a cyclic intramolecular selenurane [28]; thus, its reduction mechanism will likely differ from the one of DMSeO. This aspect certainly needs future attention, but is beyond the scope of the current investigation.

Lastly, the fully reduced chalcogenide is released after the thiolate attacks the chalconium cation. The process appears to be energetically well favored for both DMSO and DMSeO but is almost 8 kcal mol^−1^ more favored for the latter (or ca. 6 kcal mol^−1^ more favored with respect to the respective RC1). This is in line with the well-known relative instability of selenoxides with respect to sulfoxides. These results comply well with the faster reduction of selenoxides with respect to sulfoxides. Indeed, while sulfoxides require long times (hours) to induce disulfide formation, or mildly high temperature, selenoxides induce disulfide formation within minutes or even seconds at room temperature [3,5,9].

The same discussion holds true when CH_3_SeH acts as the reducing agent instead of CH_3_SH. Indeed, minimal changes in the overall mechanism are predicted from our DFT calculations (Figure 3a turquoise and Figure 3b orange). However, as expected, CH_3_SeH lowers the activation energy for U formation (e.g., while DMSO + CH_3_SH has an activation energy leading to PC1 of 21.81 kcal mol^−1^, the same step for DMSO + CH_3_SeH has an activation energy of 19.06 kcal mol^−1^). In addition, the downstream reaction steps (i.e., isomerization and PC2 formation) proceed with slightly lower activation energy when CH_3_SeH acts as the chalcogenol, leading in the very end to more stable products. However, compared to U formation, the other activation energies remain way lower. In any case, both these aspects can contribute to the advantage of Sec over Cys in MrsB selenoenzyme since they are intrinsic in the properties of either the chalcogenol itself or of the products.

### 2.3. Activation Strain Analysis

**Figure 4:**
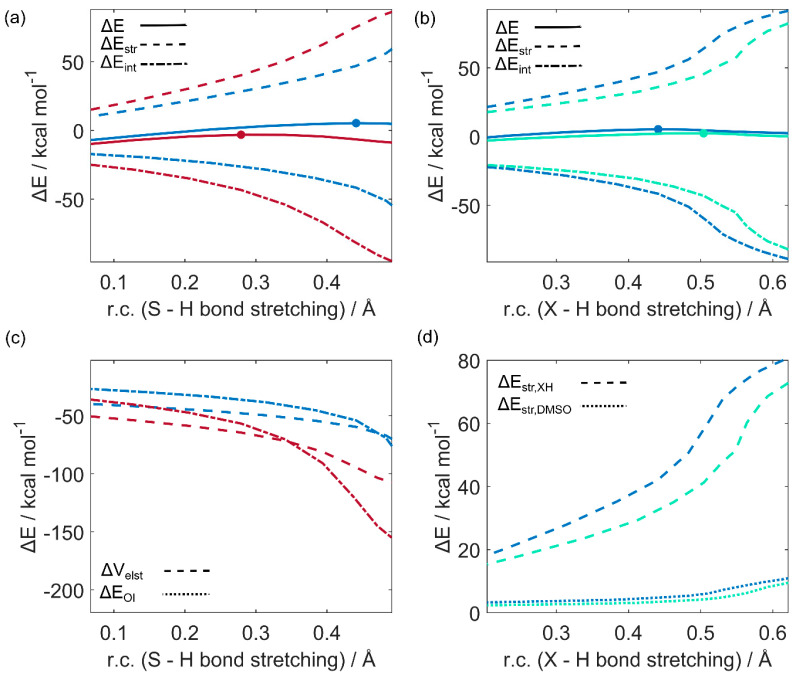
Activation strain analysis and energy decomposition analysis for three representative reactions, i.e., DMSO + CH_3_SH (dark blue); DMSeO + CH_3_SH (dark red); DMSO + CH_3_SeH (turquoise). (**a**) ASA showing the role of the chalcogen in the chalcogenoxide (DMSO vs. DMSeO). (**b**) ASA showing the role of the chalcogen in the chalcogenol, CH_3_SH vs. CH_3_SeH. (**c**) EDA showing the role of the chalcogen in the chalcogenoxide (stabilizing components only, Pauli repulsion can be found in Figure S1). (**d**) Strain decomposition analysis showing the role of the chalcogen in the chalcogenol.

Selenoxides react faster than sulfoxides [5,13,29], and selenols engage in a faster reactivity with chalcogenoxides than the lighter thiols as deduced from Msr enzymatic studies [12,15]. While our calculations reproduced and rationalized these observations, to reach a deeper understanding of these effects, DFT energy data must be translated into chemically meaningful concepts. Thus, in the spirit of “give us insight and numbers” [30], we employed the ASA/EDA approach as described in the computational methods. ASA and EDA can be rigorously applied only to gas phase calculations; luckily, the effect of the chalcogen on our reactions is qualitatively the same in both the gas phase and in water. Thus, the gas phase results can be discussed and general insight obtained with confidence. ASA/EDA plots are displayed in Figure 4. The first step of the reaction, i.e., the proton transfer from the chalcogenol to the chalcogenoxide with the concerted formation of the X–X’ chalcogen bond of PC2, was particularly investigated in detail. This step has the highest barrier in all four mechanisms.

Our systems were partitioned into two reactant-like fragments: one fragment consists of the chalcogenoxide moiety, and the other is composed by the chalcogenol and two water molecules. Focusing on the role of the chalcogen, the DMSO + CH_3_SH reaction was compared to DMSeO + CH_3_SH to understand the difference between sulfoxide and selenoxide; then, the DMSO + CH_3_SH reaction was compared to DMSO + CH_3_SeH to understand the role of the chalcogenol. The energy profiles were projected onto the critical X’–H bond stretching, which undergoes a well-defined change along the reaction.

The comparison between the ΔE(ξ) profiles for DMSO + CH_3_SH and DMSeO + CH_3_SH (Figure 4a) clearly shows that DMSeO displays an earlier TS than DMSO (r.c. ca 0.3 and 0.45 Å, respectively), and this leads to a higher activation energy when the former is involved. ASA highlights that this is due to a more stabilizing $\Delta E_{int\;}\left(\xi \right)$ along the whole r.c. for DMSeO, but particularly in the surrounds of the TSs. EDA (Figure 4c) pinpoints how both a stronger electrostatic and orbital interaction between the two fragments is responsible for the more stabilizing $\Delta E_{int\;}\left(\xi \right)$ of DMSeO. Indeed, both $\Delta V_{elst\;}\left(\xi \right)$ and $\Delta E_{OI\;}\left(\xi \right)$ are more stabilizing for DMSeO than for DMSO. While, in magnitude, $\Delta V_{elst\;}\left(\xi \right)$ appears to play a slightly more significant role in the beginning of the reaction, $\Delta E_{OI\;}\left(\xi \right)$ soon becomes more and more determinant as the S–H bond is broken and an X–X’ bond is formed. Thus, we will not try to assert one single predominant contribution, since both factors contribute to some extent to the enhanced reactivity of DMSeO.

**Figure 5:**
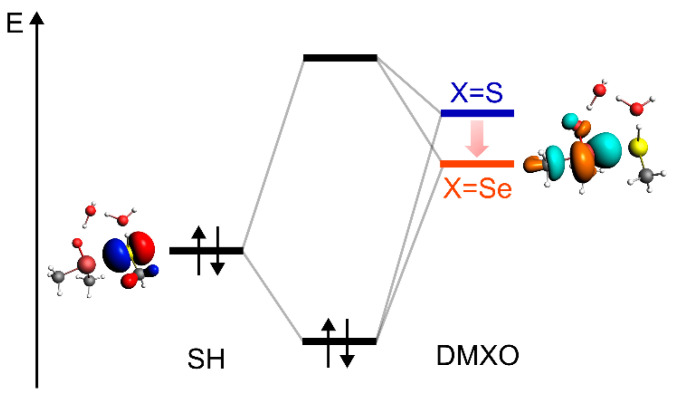
Schematic qualitative molecular orbital diagram for the orbital interaction between the HOMO of CH_3_SH (on the **left**) and the LUMO of DMXO (on the **right**). The lower energy of DMSeO LUMO is emphasized with an arrow pointing downward. The calculated Kohn-Sham molecular orbitals for DMSeO + SH fragments are represented on the side of the diagram (isodensity value 0.05; r.c. = 0.341).

The larger $\Delta V_{elst\;}\left(\xi \right)$ effect is interpreted as a consequence of the more charge separated Se–O bond, when compared to the S–O bond, which leads to a more negative potential on the oxygen atom and to a higher positive potential on the chalcogen nucleus of DMSeO [13,27]. This results in a combined stronger electrostatic interaction with the proton transferred from water, as well as with the thiolate. Finally, the more stabilizing $\Delta E_{OI\;}\left(\xi \right)$ is ascribed to the low-lying LUMO of DMSeO when compared to DMSO, which can more easily interact with the HOMO of CH_3_SH (Figure 5). Thus, DMSeO takes part in the reaction as the electrophile, and the thiol/thiolate as the nucleophile, resulting in a 1,2 nucleophilic addition reaction.

Conversely, the comparison of the ASA plots for DMSO + CH_3_SH and DMSO + CH_3_SeH (Figure 4b) shows a quite different picture. Specifically, while the ΔE(ξ) profile is always stabilized when CH_3_SeH acts as the nucleophile as compared to CH_3_SH, CH_3_SeH reaches the transition state later along the r.c. Despite this, DMSO + CH_3_SeH reaction is characterized by a significantly lower $\Delta E_{str\;}\left(\xi \right)$ profile, which is ultimately responsible for the faster reactivity of selenols as compared to thiols. Since the chalcogenoxide is the same in the two reactions, it is reasonable to assume this effect to be directly related to the ease by which X’–H distorts. However, to provide a quantitative discussion, the overall $\Delta E_{str\;}\left(\xi \right)$ was further decomposed into the separate contributions of the two fragments (Figure 4d). As expected, DMSO gives a minimal (and very similar for the two reactions) contribution to the overall $\Delta E_{str\;}\left(\xi \right)$; conversely, the X’–H bond breaking provides the determinant contribution to $\Delta E_{str\;}\left(\xi \right)$, with CH_3_SeH undergoing a less destabilizing distortion than CH_3_SH. This physico-chemical mechanism is, indeed, quite general, and was explored in detail by Dalla Tiezza et al. in model systems of GPx [25], and complies with the well-known increased acidity of the heavier chalcogenols as compared to the lighter ones.

Overall, the effect of the chalcogen on the chalcogenoxide is electrophilic in nature; conversely, CH_3_SeHs are better nucleophiles because they undergo an easier heterolytic dissociation, shuttling their proton to the chalcogenoxide and nucleophilically binding it.

### 2.4. Direct vs. Indirect Reduction

As a last goal, we present a more exhaustive discussion about the final step of the reduction mechanism (Scheme 3c). As previously anticipated, all the attempts to optimize transition state structures in the gas phase for both processes (i.e., direct and indirect, Path I and II, respectively, in Scheme 3) failed, likely because the negatively charged thiolate and the positively charged chalconium cation spontaneously recombine. Thus, to provide a quantitative discussion, an analysis was carried out by optimizing the geometries in the presence of the solvation field, i.e., at COSMO-ZORA-BLYP-D3(BJ)/TZ2P. Then, single-points in solvent were performed using the M06 functional combined to TZ2P basis set in order to have energy values consistent with the rest of the points along the mechanism.

**Figure 6:**
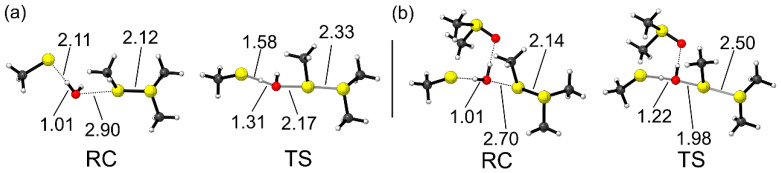
Representative structures for the indirect reduction mechanism of DMSO: (**a**) without an extra DMSO stabilizing H_2_O; (**b**) with an extra DMSO stabilizing H_2_O. Interatomic distances (in Å) that undergo relevant changes along the reaction are indicated. Level of theory: COSMO-ZORA-BLYP-D3(BJ)/TZ2P.

Interestingly, no transition state was located for the direct pathway. Even in the presence of the solvent field, the thiolate and the chalconium cation recombined without any appreciable activation energy at our level of theory when starting from a linear X–X’–S arrangement. Balta et al. [20] located a low energy TS for the reduction of DMSO. However, it is worth noticing that in their case the process was modeled with a non-linear S–S–S arrangement, which is expected to disfavor an S_N_2-like process. Since the transition state they reported was already low in energy (ΔE^‡^ ca. 2 kcal mol^−1^), we confidently believe that with a linear arrangement the process should be almost instantaneous also at their level of theory. Conversely, water activation by the thiolate, which leads to the formal OH^−^ attack to the chalcogenurane appears to be an activated process (Figure 6).

The activation energy of the process is modulated by both the chalcogen on the chalcogenoxide and by the chalcogen on CH_3_X’H, with a larger influence of the latter. Indeed, changing the chalcogen from DMSO to DMSeO affects ΔE^‡^_indir_ by roughly 3 kcal mol^−1^. Conversely, changing the chalcogen on CH_3_X’H leads to a ΔE^‡^ of more than 7 kcal mol^−1^ at our level of theory (Table 1).

In particular, while changing X from S to Se on the chalcogenoxide leads to an increase in the activation energy required to break the X–X’ bond, changing S with Se on CH_3_X’H leads to a sharp decrease in the activation energy. This is not completely unexpected, since X’ acts as the central atom in the S_N_2-like process (reaction 3 in Scheme 3). Thus, the more electrophilic central Se nucleus is expected to provide a kinetic advantage in the reaction. Nevertheless, in all cases under investigation, indirect reductions involving Se are energetically more favored than the correspondent ones with S, but all are less favored than the corresponding direct reduction. Thus, for reactions occurring in solution, the direct reduction appears to be both kinetically and thermodynamically favored, regardless of the chalcogens. Although tempting, the discussion cannot be safely translated into the enzymatic context: indeed, the formation of a sulfenic acid has been confidently assessed at least in some Msrs proteins [16,21]. Thus, it is possible that geometric constraints within the enzyme architecture somewhat suppress the direct reduction and that water activation by other residues promotes sulfenic acid formation [31,32]. Instead, in our model, which more accurately describes the reduction in solution, only the thiolate can activate H_2_O. In any case, also in this step, Sec95 could provide an advantage over Cys95 since Se would participate in the selenenic acid formation as the central atom of the S_N_2 process as described above.

## 3. Methods and Materials

All density functional theory (DFT) calculations were carried out using ADF2019 [33,34]. For geometry optimization of all minima and transition states, BLYP density functional was employed, combined with the Grimme D3 dispersion correction and Becke–Johnson damping [35,36,37,38,39]. The TZ2P basis set (triple-ζ with two sets of polarization functions on each atom) with the small frozen core approximation was used in all calculations. Scalar relativistic effects were included at the zeroth-order relativistic approximation (ZORA), as implemented in ADF [40]. This level of theory, i.e., ZORA-BLYP-D3(BJ)/TZ2P, was benchmarked and found adequate for the structural description of organochalcogenides, with the advantage of not being too computationally expensive to perform extensive mechanistic investigations [41]. To ascertain the nature of each optimized geometry, frequency calculations were performed in gas phase at the same level of the theory. All minima displayed only positive frequencies, while transition states displayed only one negative frequency associated to the nuclear motion along the reaction coordinate. Additionally, for selected transition states (TS), the minimum energy path connecting the TS to the two closest minima was obtained via an intrinsic reaction coordinate (IRC) procedure as implemented in the ADF software [42]. All optimized cartesian coordinates are available in the supplementary materials (Tables S3 and S4).

To further increase the accuracy of the energy description, single-point calculations were performed using M06 functional combined with an all-electron TZ2P basis set [43]. The combination of a geometry optimization performed with a validated GGA and single-points performed with M06 meta-hybrid functional proved to be one of the best approaches to reproduce the energetics of other organochalcogen reactions [27] as well as more common key organic reactions (S_N_2 and E2 mechanisms) [44] when compared to high-level ab initio calculation results. Additionally, M06 functional is one of the top scoring DFT approaches to deal with non-covalent interactions such as chalcogen bonds [45]. In any case, to provide further confidence to the energetics investigated in this work, activation energies for DMSO and DMSeO reduction obtained at ZORA-M06/TZ2P-ae//ZORA-BLYP-D3(BJ)/TZ2P were compared to those obtained at the DLPNO-CCSD(T)/aug-cc-pVTZ-DK level of theory. DLPNO-CCSD(T) calculations were performed with Orca 4.2.0 software [46,47,48] (Table S2). The effect of implicit solvation was included with the M06 functional using the COSMO model of solvation as implemented in ADF [49]. Water was chosen as the solvent to account for the effect of a strongly polar environment, which is the most likely to significantly affect the energetics. All energetics discussed in the main text were thus computed at COSMO-ZORA-M06/TZ2P-ae//ZORA-BLYP-D3(BJ)/TZ2P level of theory. For simplicity, this level of theory is from now on labeled as COSMO-M06//BLYP-D3(BJ).

To provide a deeper insight into the effect of the chalcogen (S vs. Se) on the chalcogenoxide and the chalcogenol, the activation strain model (ASM) of chemical reactivity was applied to step 1 in Scheme 2 employing the program PyFrag 2019 [50]. The ASM is a fragment-based approach which, when two chemically useful fragments are chosen (i.e., usually the reactants), allows to partition any energy ΔE along a (suitable) reaction coordinate (ξ) into two terms, that is, deformation, or strain, energy ($\Delta E_{str}$) and interaction energy ($\Delta E_{int}$).

(1) $$\Delta E\left(\xi \right)=\Delta E_{str\;}\left(\xi \right)+\Delta E_{int\;}\left(\xi \right)$$

These two terms account for the energy required to deform the reactants from their equilibrium geometry to the geometry they display at ξ, and the actual instantaneous interaction between the two distorted fragments, respectively. Based on this definition, $\Delta E_{str}$ is destabilizing in nature, while $\Delta E_{int}$ is stabilizing. This first decomposition is commonly referred to as activation strain analysis (ASA). In fact, $\Delta E_{int\;}\left(\xi \right)$ can be further decomposed into chemically meaningful terms according to the energy decomposition analysis (EDA) scheme implemented in ADF [51,52,53,54].

(2) $$\Delta E_{int\;}\left(\xi \right)=\Delta V_{elst\;}\left(\xi \right)+\Delta E_{Pauli\;}\left(\xi \right)+\Delta E_{OI\;}\left(\xi \right)$$

Within this framework, $\Delta E_{int\;}\left(\xi \right)$ can be expressed as the sum of three terms: $\Delta V_{elst\;}\left(\xi \right),$ the semi-classical electrostatic interaction between the unrelaxed electron densities of the two fragments; $\Delta E_{Pauli\;}\left(\xi \right)$, the so-called Pauli repulsion, i.e., the destabilizing two-centers four-electrons interaction; and $\Delta E_{OI\;}\left(\xi \right)$, called the orbital interaction, i.e., the stabilizing two-centers two-electrons interaction.

Since both ASA and EDA can be rigorously applied only to gas phase electronic energies, M06//BLYP-D3(BJ) calculations were employed, i.e., results obtained without the COSMO solvation model. Further details are given in Section 2.3. For the sake of clarity, only electronic energies are discussed in the main text. Since the interesting comparison is between structurally analogous reactions, entropy corrections are expected to leave the trends unaffected. Indeed, Gibbs free activation and reaction energies for the key mechanistic step followed essentially analogous trends for the effect of the chalcogen on the chalcogenoxide and the chalcogenol (DMSO vs. DMSeO + CH_3_SH and DMSO + CH_3_SH vs. DMSO + CH_3_SeH, respectively, in Table S1).

## 4. Conclusions

In this work we investigated the molecular mechanism for the reduction of DMSO and DMSeO to sulfide and selenide by two equivalents of methyl thiol or by a mixed methyl selenol-methyl thiol reducing system. While the reduction of DMSO by methyl thiol has already been studied in the past [1,20], little attention was devoted to the reactions in which other chalcogens act as oxidizing or reducing agents. We proved that DMSeO is a better oxidizing agent mainly because the selenurane, which is a key intermediate, is more stable and is formed more easily than the analogous sulfurane. Activation strain analysis proved that this is related to the stronger electrophilic nature of DMSeO when compared to DMSO.

Selenium also provides a kinetic advantage when it acts as the reducing selenol. In this case, the increased acidity of the selenol provides the decisive contribution to the reaction, since the Se–H bond is broken more easily than S–H, analogously to what was found for the mechanism of GPx and related molecular models [23,25]. Overall, these results provide a comprehensive theoretical physico-chemical look at the title reaction that complements the available experimental literature.

The system properly investigated here describes the reaction as it occurs in solution. However, the effect of the chalcogenol can at least be qualitatively used to interpret the kinetic competence of the human selenoenzyme MsrB1, in which a selenocysteine is present as catalytic residue. To the best of our knowledge, this is the first time that the kinetic advantage of Sec over Cys in MsrB catalysis has been dealt with in silico, providing a preliminary theoretical rationale to the experimental data. Importantly, this outcome is another puzzle piece in the redox biology topic on the role of selenium rather than sulfur, which is still an open problem [13].

Future investigations focusing on the role of Sec in MsrB catalysis in more realistic enzymatic models are expected to provide a more complete insight into one of the few selenoenzymes with a known function especially when comparison with molecular model mechanisms, such as those provided in this work, are available as a starting point.

## Figures and Tables

**Table 1:** Activation and reaction energies ^1^ (kcal mol^−1^) for the direct and indirect evolution of the chalconium cation toward disulfide/selenyl sulfide or sulfenic/selenenic acid, respectively ^2^.

	ΔE^‡^_dir_	ΔE_r,dir_	ΔE^‡^_indir_	ΔE_r,indir_
DMSO + CH_3_SH	-	−25.62	14.48 (12.75) ^3^	0.46
DMSeO + CH_3_SH	-	−33.02	17.80	−6.94
DMSO + CH_3_SeH	-	−30.68	6.94	−5.39
DMSeO + CH_3_SeH	-	−38.03	9.98	−12.79

^1^ ΔE^‡^ are computed with respect to the closest minimum (i.e., a reactant complex-like structure), while ΔE_r_ are computed with respect to the free reactants (DMXO, CH_3_X’H, and H_2_O). ^2^ ΔE^‡^_indir_ is computed at COSMO-M06//COSMO-BLYP-D3(BJ) as explained in the discussion. ^3^ Between parentheses, indirect transition state with one extra DMSO coordinating H_2_O. Level of theory COSMO-M06//BLYP-D3(BJ).

## Data Availability

All data required supporting the results are available in the main text or in the Supplementary Materials.

## Supplementary Materials

The supporting information can be downloaded at: https://www.mdpi.com/article/10.3390/ijms24097754/s1.
