# Supplementary material for: The Key Role of Chalcogenurane Intermediates in the Reduction Mechanism of Sulfoxides and Selenoxides by Thiols Explored In Silico
Source: Int J Mol Sci. 2023 Apr 24;24(9):7754. doi: 10.3390/ijms24097754 (PMC10178455; doi:10.3390/ijms24097754)
Supplement: Supplementary file 1 [file ijms-24-07754-s001.zip › ijms-2331059-supplementary.pdf]

SUPPORTING INFORMATION

The Key Role of Chalcogenurane Intermediates In the Reduction Mechanism of Sulfoxides and Selenoxides by Thiols Explored *In Silico*

Andrea Madabeni,[1] Laura Orian [1,*]

[1] Dipartimento di Scienze Chimiche, Università degli Studi di Padova, Via Marzolo 1, 35131 Padova, Italy.
*Author to whom correspondence should be addressed.

Tables of Contents

Table S1: Activation and reaction[1] Gibbs free energies (kcal mol-1) for the reactions DMSO+SH, DMSeO+SH and DMSO+SeH (see main text). Level of theory: COSMO-M06 // BLYP-D3(BJ).[2] 5

Table S2: DLPNO-CCSD(T)Activation energies (kcal mol^{-1}) and M06 // BLYP-D3(BJ) in parenthesis..................... 5

Figure S1: Pauli Repulsion plot for DMSO+SH (blue) vs DMSeO+SH (dark red). 6

Scheme S1. Catalytic cycle for MrsA as hypothesized by Levine *et al*. 7

Table S3: Cartesian coordinates (Å), energies (kcal mol^{-1}) and imaginary frequencies (cm^{-1}) of the optimized structures. Level of theory: COSMO-ZORA-M06/TZ2P-ae // ZORA-BLYP-D3(BJ). 7

Table S4: Cartesian coordinates (Å), energies (kcal mol^{-1}) and imaginary frequencies (cm^{-1}) of the optimized structures for sulfenic and selenenic acid formation. Level of theory: COSMO-ZORA-M06/TZ2P-ae // COSMO-ZORA-BLYP-D3(BJ). 14

Table S1: Activation and reaction[1] Gibbs free energies (kcal mol-1) for the reactions DMSO+SH, DMSeO+SH and DMSO+SeH (see main text). Level of theory: COSMO-M06 // BLYP-D3(BJ).[2]

	$\Delta G^{\ddagger}_{TS1}$	ΔG_{PC1}	$\Delta G^{\ddagger}_{TS1-2}$	ΔG_{U}	$\Delta G^{\ddagger}_{TS2}$ [3]	ΔG_{PC2}	ΔG_{r}
DMSO+CH_3SH	21.65	21.70	24.72	17.45	1.28 (26.80)	-6.79	-22.5
DMSeO+ CH_3SH	18.76	11.53	15.82	0.77	2.74(8.48)	-0.25	-29.04
DMSO+ CH_3SeH	19.87	19.15	22.12	16.12	-0.67(22.42)	-10.81	-27.25

[1]Activation energies and energies of PC and U are relatives to the *relative* RC. The reaction Gibbs free energy is relative to the free reactants. [2]The contribution of solvation is taken into account at the M06 level, while thermodynamics corrections are taken into account at the BLYP-D3(BJ) level of theory.[3] The energy in parenthesis is with respect to RC1.

A discussion based on Gibbs free energies provides qualitatively similar discussions. Particularly, (i) TS1 is always the step with the highest activation energy with respect to the closest RC, even if for DMSO TS2 is even higher on PES (but has a lower activation energy with respect to the closest minimum). (ii) changing the chalcogen from S to Se on DMXO or on CH_3XH leads to a reduction of a couple of kcal mol^{-1} in the activation energy of this step. (iii) The energy of U with respect to RC1 is the most remarkable change in the mechanism when changing the substrate from DMSO to DMSeO, with DMSeO having a much more stable intermediate U. (iv) The overall thermodynamics is more favorable for DMSeO than for DMSO, and more favorable for CH_3SeH than for CH_3SH.

Table S2: DLPNO-CCSD(T)Activation energies (kcal mol^{-1}) and M06 // BLYP-D3(BJ) in parenthesis. Activation and reaction energies are referred to the respective RC.

	$\Delta E^{\ddagger}_{TS1}$	$\Delta Er, U$	$\Delta E^{\ddagger}_{TS2}$	$\Delta Er, PC2$
DMSO+CH_3SH	25.13(25.23)	12.97 (12.94)	3.66(4.34)	-1.64(-1.73)
DMSeO+ CH_3SH	21.42(20.15)	-3.95 (-5.06)	3.29(3.01)	-2.67(-0.15)

DLPNO-CCSD(T) single point calculations were done on BLYP-D3(BJ) optimized geometries, employing the Tight PNO criteria and the aug-cc-pVTZ-DK basis set with Douglas−Kroll−Hess (DKH) scalar relativistic Hamiltonian for all atoms.

While this works does not have the ambition to provide a rigorous and extensive benchmark for organochalcogen chemistry or organic reactivity in general (see main text for ref. about published benchmark in the field), a couple of activation and reaction energies were recomputed employing a high-level *ab initio* method, i.e. DLPNO-CCSD(T) with a large relativistic basis set aug-cc-pVTZ-DK, DKH relativistic approximation and TightPNO criteria for the DLPNO approximation, as recommended. Tight criteria for SCF convergence have been employed. The combination of a geometry optimization with a GGA functional, plus single point with a metahybrid (M06 or M06-2X) functional was found to be one of the best approaches to tackle organochalcogenides' reactivity in the past by some of us (ref. 37 in the main text) and to treat other organic reactions and non-covalent interactions by others (ref. 38 and 39 in the main text). Thus, here,

DLPNO-CCSD(T) single points are not intended for extensive benchmarking purposes (the method is already validated and consolidated) but just as a further safety check to confirm that the chosen level of theory properly reproduces the main characteristics of the potential energy surfaces under investigation. The close agreement (in some cases, even within fractions of kcal mol^{-1}) of M06 // BLYP-D3(BJ) with the DLPNO-CCSD(T) // BLYP-D3(BJ) energies, gives us further confidence in the chosen methodology, especially since we are interested in relative trends and not in absolute values. The only small but noticeable disagreement between DLPNO-CCSD(T) and DFT is in the relative energy order of PC2 for DMSO and DMSeO. However, the energy difference of PC2 for DMSO and DMSeO, computed with DFT and with CCSD(T) is of roughly 1 – 1.5 kcal mol^{-1}, very close at both levels of theory. Thus, both methods reach the same conclusion: in gas phase, the step remains weakly exergonic (both for DMSO and DMSeO) and with a low activation energy, especially when compared to TS1.

Figure S1: Pauli Repulsion plot for DMSO+SH (blue) vs DMSeO+SH (dark red).

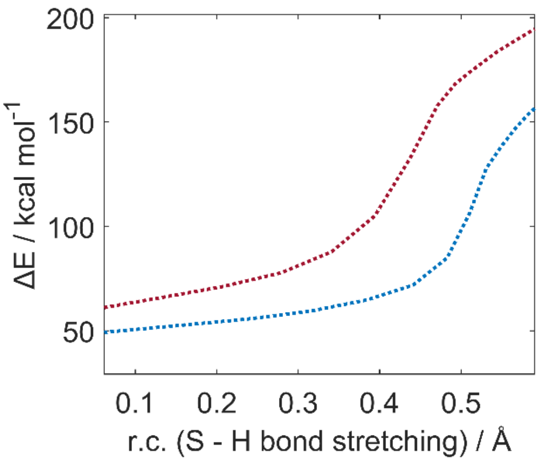

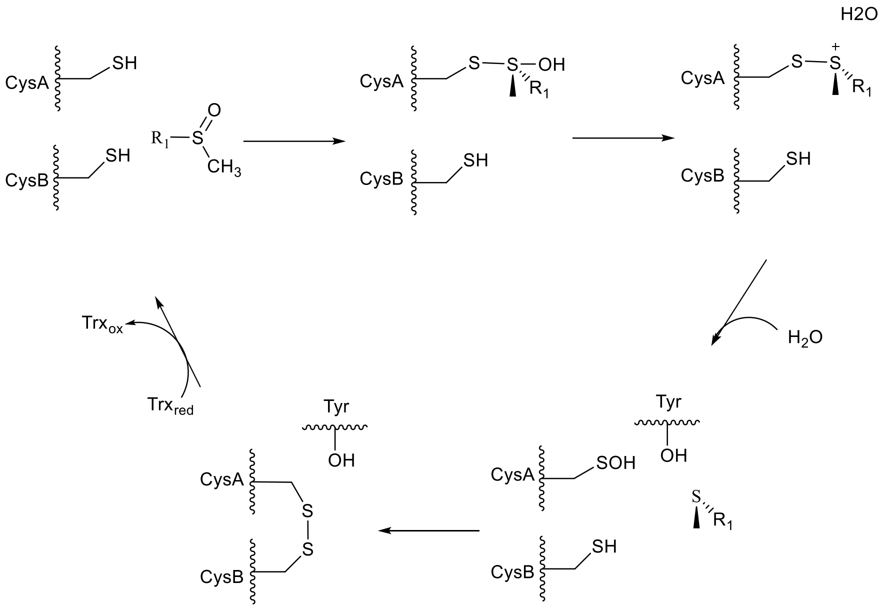

Scheme S1. Catalytic cycle for MrsA as hypothesized by Levine *et al*.

Table S3: Cartesian coordinates (Å), energies (kcal mol^{-1}) and imaginary frequencies (cm^{-1}) of the optimized structures. Level of theory: COSMO-ZORA-M06/TZ2P-ae // ZORA-BLYP-D3(BJ).

Reactants

DMSO

E= -1411.95

Nimag=0

C	0.491900000	0.025300000	-1.369600000
S	-0.562900000	0.683500000	0.000000000
O	-1.837300000	-0.123900000	0.000000000
C	0.491900000	0.025300000	1.369600000
H	-0.017300000	0.281400000	-2.301900000
H	1.476900000	0.501600000	-1.331100000
H	0.568200000	-1.061200000	-1.263900000
H	1.476900000	0.501600000	1.331100000
H	-0.017300000	0.281400000	2.301900000
H	0.568200000	-1.061200000	1.263900000

DMSeO

E= -1382.83

Nimag=0

C	0.524700000	0.029300000	-1.465100000
Se	-0.638900000	0.784700000	0.000000000
O	-1.990500000	-0.206600000	0.000000000
C	0.524700000	0.029300000	1.465100000
H	0.035100000	0.280700000	-2.407700000
H	1.519500000	0.479600000	-1.403400000
H	0.554900000	-1.051900000	-1.316100000
H	1.519500000	0.479600000	1.403400000
H	0.035100000	0.280700000	2.407700000
H	0.554900000	-1.051900000	1.316100000

CH_3SH

E= -763.49

Nimag=0

S	1.887600000	-2.878300000	-1.006700000
C	1.770200000	-3.452300000	0.744300000
H	1.717900000	-2.605000000	1.431800000
H	0.912000000	-4.113800000	0.883100000
H	2.688700000	-4.012900000	0.936700000
H	0.699000000	-2.232100000	-1.040200000

CH3SeH

E= -740.39

Nimag=0

Se	2.952600000	-0.946100000	0.000000000
C	2.156200000	-2.781100000	0.000000000
H	1.559300000	-2.926900000	0.900600000
H	1.559300000	-2.926900000	-0.900600000
H	3.006400000	-3.466200000	0.000000000
H	1.644800000	-0.247200000	0.000000000

Products

Dimethyl disulfide

E= -1333.10

C	-0.459100000	-0.865000000	-1.242700000
S	-0.627900000	0.858200000	-0.603300000
H	-1.455100000	-1.314200000	-1.160000000
H	-0.145000000	-0.848700000	-2.288700000
H	0.249300000	-1.435700000	-0.637000000
S	1.254100000	1.672000000	-0.916100000
C	2.196800000	1.154800000	0.584000000
H	1.698200000	1.518500000	1.485300000
H	2.307000000	0.068100000	0.617200000
H	3.184900000	1.619400000	0.492000000

Dimethyl selenyl sulfide
E= -1315.06
Nimag=0

C	-1.305200000	2.240800000	0.206000000
S	0.190900000	1.358700000	0.831500000
H	-1.929500000	2.580800000	1.035700000
H	-1.014800000	3.083000000	-0.425800000
H	-1.858100000	1.504800000	-0.389000000
C	0.393200000	2.829100000	3.725100000
H	0.924400000	3.532100000	4.374100000
H	-0.653400000	3.120100000	3.628800000
H	0.490000000	1.810700000	4.102300000
Se	1.292600000	2.945600000	1.939400000

Dimethyl sulfide
E= -1208.11
Nimag=0

C	-1.394400000	0.000000000	-0.087300000
S	0.000000000	0.000000000	1.102700000
C	1.394400000	0.000000000	-0.087300000
H	-2.315500000	0.000000000	0.502300000
H	-1.370200000	0.896800000	-0.715400000
H	-1.370200000	-0.896800000	-0.715400000
H	1.370200000	0.896800000	-0.715400000
H	2.315500000	0.000000000	0.502300000
H	1.370200000	-0.896800000	-0.715400000

Dimethyl selenide
E= -1186.39
Nimag=0

C	-1.480900000	0.000000000	-0.103400000
Se	0.000000000	0.000000000	1.226200000
C	1.480900000	0.000000000	-0.103400000
H	-2.412600000	0.000000000	0.467000000
H	-1.422400000	0.899300000	-0.720600000
H	-1.422400000	-0.899300000	-0.720600000
H	1.422400000	0.899300000	-0.720600000
H	2.412600000	0.000000000	0.467000000
H	1.422400000	-0.899300000	-0.720600000

Water
E= -423.34
Nimag=0

O	0.000000000	0.000000000	-0.634700000
H	0.000000000	0.768800000	-0.039000000
H	0.000000000	-0.768800000	-0.039000000

RCs

DMSO+SH
RC1
E= -3031.25
Nimag=0

C	1.362700000	0.854000000	-0.639000000
S	-0.224400000	0.918000000	0.278700000
O	-1.309700000	0.620000000	-0.753900000
C	0.003300000	-0.654300000	1.208800000
H	1.372600000	1.739800000	-1.276600000
H	2.187600000	0.896200000	0.079000000
H	1.390000000	-0.067300000	-1.228000000
H	0.861700000	-0.550500000	1.880100000
H	-0.911600000	-0.807100000	1.786300000
H	0.148500000	-1.472300000	0.497200000
C	1.416800000	4.167700000	1.256600000
H	1.105700000	3.217400000	1.699300000
H	2.481600000	4.128000000	1.011900000
H	1.241200000	4.979900000	1.965800000
S	0.511000000	4.467100000	-0.321300000
H	-0.735900000	4.362200000	0.225000000
O	-2.381400000	3.887900000	1.315500000
H	-3.003500000	4.588200000	1.573000000
H	-2.873900000	3.326900000	0.657300000
O	-3.511000000	2.199800000	-0.552900000
H	-3.658400000	2.599400000	-1.426200000
H	-2.728100000	1.592500000	-0.676200000

RC2
E=-4339.61
Nimag=0

C	-2.199500000	2.009100000	0.569100000
S	-1.888600000	0.208500000	0.398600000
O	-0.053600000	0.570200000	1.001900000
C	-1.295900000	0.080400000	-1.336000000
O	1.011200000	-1.974600000	0.393300000
S	2.513600000	-2.193100000	0.274200000
C	3.023600000	-1.524900000	-1.363900000
C	3.326300000	-0.883800000	1.284000000
S	-4.118400000	-0.241000000	-0.281900000
C	-4.710800000	-0.628200000	1.415600000
S	1.938300000	2.190900000	-0.863300000
C	2.520700000	3.363900000	0.438700000
H	-5.573500000	-1.294900000	1.330300000
H	-3.922800000	-1.145700000	1.975400000
H	-5.010000000	0.276800000	1.953500000
H	-2.764700000	2.134800000	1.494800000
H	-2.800300000	2.336700000	-0.278000000
H	-1.233900000	2.509900000	0.628100000
H	-0.681500000	0.957300000	-1.541400000
H	-0.684600000	-0.823900000	-1.363200000
H	-2.143600000	0.008600000	-2.017100000
H	0.340000000	-0.334800000	0.949600000
H	2.518200000	-2.135300000	-2.116400000
H	2.727700000	-0.473900000	-1.434500000
H	4.109100000	-1.633300000	-1.458100000
H	3.035400000	-1.066800000	2.321100000
H	4.410300000	-0.983400000	1.169200000
H	2.979400000	0.095500000	0.943100000
H	1.063000000	1.482700000	-0.045400000
H	3.246400000	4.032500000	-0.030800000
H	1.686100000	3.954000000	0.824800000
H	3.004100000	2.826500000	1.258900000

DMSO+SeH
RC1
E= -3007.85
Nimag=0

C	1.378800000	0.835300000	-0.638200000
S	-0.224700000	0.951900000	0.245800000
O	-1.292900000	0.637200000	-0.799400000

C	-0.038900000	-0.593400000	1.229400000
H	1.414500000	1.700200000	-1.303400000
H	2.190800000	0.887500000	0.093700000
H	1.403000000	-0.103800000	-1.198300000
H	0.808000000	-0.480800000	1.913800000
H	-0.966800000	-0.713100000	1.793600000
H	0.106800000	-1.436300000	0.547500000
C	1.500600000	4.170800000	1.261700000
H	1.158100000	3.213600000	1.658200000
H	2.556000000	4.115900000	0.987100000
H	1.343200000	4.965200000	1.991600000
Se	0.492100000	4.580300000	-0.414100000
H	-0.836300000	4.379100000	0.239500000
O	-2.424900000	3.764800000	1.387400000
H	-3.052800000	4.447700000	1.676200000
H	-2.904600000	3.246600000	0.686200000
O	-3.511900000	2.192000000	-0.604900000
H	-3.631300000	2.637800000	-1.459900000
H	-2.722000000	1.595000000	-0.733300000

RC2
E= -4318.80
Nimag=0

C	-2.192700000	2.083200000	0.511900000
S	-1.893200000	0.279500000	0.355300000
O	-0.047000000	0.632800000	1.001000000
C	-1.268300000	0.153300000	-1.369300000
O	0.957800000	-1.942800000	0.398900000
S	2.453900000	-2.204500000	0.287900000
C	2.991900000	-1.549500000	-1.346900000
C	3.298600000	-0.920100000	1.303300000
Se	-4.259900000	-0.224600000	-0.352800000
C	-4.736900000	-0.782500000	1.503500000
S	1.995500000	2.188900000	-0.851000000
C	2.570400000	3.386100000	0.432200000
H	-5.509100000	-1.550900000	1.431400000
H	-3.845500000	-1.203700000	1.977400000
H	-5.107200000	0.070400000	2.075100000
H	-2.750100000	2.219600000	1.440600000
H	-2.796300000	2.412500000	-0.332500000
H	-1.221600000	2.575300000	0.559000000
H	-0.612800000	1.006400000	-1.546100000
H	-0.696400000	-0.776300000	-1.397500000
H	-2.100400000	0.127500000	-2.072300000
H	0.328700000	-0.280000000	0.956000000
H	2.473600000	-2.144400000	-2.102900000
H	2.726400000	-0.490600000	-1.417800000
H	4.074400000	-1.688500000	-1.435300000
H	2.996400000	-1.095200000	2.338500000
H	4.379900000	-1.051600000	1.194800000
H	2.982900000	0.069300000	0.961000000
H	1.096700000	1.509300000	-0.031500000
H	3.316900000	4.030300000	-0.038600000
H	1.737900000	3.999600000	0.785200000
H	3.027100000	2.862000000	1.275800000

DMSeO+SH
RC1
E= -3003.68
Nimag=0

C	1.424000000	0.998200000	-0.475500000
Se	-0.346100000	1.135300000	0.440100000
O	-1.429400000	0.755700000	-0.811000000
C	-0.162800000	-0.640200000	1.361000000
H	1.499800000	1.888600000	-1.099600000
H	2.210300000	0.975700000	0.283900000
H	1.403700000	0.083200000	-1.070000000
H	0.681900000	-0.596800000	2.054500000
H	-1.100300000	-0.806100000	1.894600000
H	-0.015400000	-1.392800000	0.584300000
C	1.080000000	4.564400000	1.302300000
H	0.802600000	5.432300000	1.904800000
H	0.888700000	3.647300000	1.867600000
H	2.142700000	4.624300000	1.053700000
S	0.154300000	4.550300000	-0.291900000
H	-1.079600000	4.395600000	0.273100000
O	-2.671700000	3.760400000	1.340900000
H	-3.333200000	4.381600000	1.687600000
H	-3.140400000	3.243300000	0.626300000
O	-3.682500000	2.193300000	-0.654000000
H	-3.803600000	2.633100000	-1.511900000
H	-2.856900000	1.626300000	-0.764000000

RC2
E= -4331.62
Nimag=0

C	-2.134946000	2.064439000	0.561609000
Se	-1.900780000	0.092013000	0.534672000
O	0.034546000	0.494548000	1.202949000
C	-1.196493000	-0.130746000	-1.311332000
O	1.135611000	-2.013362000	0.419236000
S	2.637001000	-2.160670000	0.208514000
C	3.019785000	-1.432387000	-1.438683000
C	3.447893000	-0.837734000	1.201938000
S	-4.198607000	-0.292840000	-0.308679000
C	-4.963377000	-0.517927000	1.354260000
S	1.773376000	2.196220000	-0.779353000
C	2.469810000	3.338371000	0.493647000
H	-5.919907000	-1.029212000	1.213400000
H	-4.325667000	-1.142450000	1.989923000
H	-5.143822000	0.443538000	1.844742000
H	-2.549496000	2.305503000	1.541883000
H	-2.836055000	2.315240000	-0.232051000
H	-1.146723000	2.501609000	0.427356000
H	-0.715630000	0.808273000	-1.584224000
H	-0.467045000	-0.937442000	-1.229353000
H	-2.023288000	-0.382922000	-1.973629000
H	0.457573000	-0.391746000	1.109497000
H	2.499531000	-2.048061000	-2.176643000
H	2.673634000	-0.394511000	-1.464944000
H	4.101697000	-1.488822000	-1.597538000
H	3.231534000	-1.058979000	2.249689000
H	4.526103000	-0.880904000	1.018033000
H	3.032995000	0.130448000	0.909282000
H	1.007426000	1.430631000	0.107739000
H	3.102692000	4.059989000	-0.028725000
H	1.667674000	3.871060000	1.010258000
H	3.074651000	2.789533000	1.220727000

DMSeO+SeH
RC1
E= -2980.15

Nimag=0

C	1.511600000	0.775100000	-0.656500000
Se	-0.211700000	1.050400000	0.315700000
O	-1.358800000	0.723800000	-0.893200000
C	-0.117600000	-0.717300000	1.266200000
H	1.626300000	1.646300000	-1.301600000
H	2.321400000	0.713100000	0.075700000
H	1.408300000	-0.147000000	-1.231000000
H	0.753100000	-0.719700000	1.928200000
H	-1.043900000	-0.806500000	1.836300000
H	-0.051400000	-1.492700000	0.500900000
C	1.506500000	4.359600000	1.243600000
H	1.257400000	3.416600000	1.734300000
H	2.559200000	4.370700000	0.954900000
H	1.288800000	5.200700000	1.902100000
Se	0.450100000	4.530600000	-0.443600000
H	-0.858200000	4.346300000	0.255000000
O	-2.395100000	3.654500000	1.413200000
H	-3.012400000	4.287000000	1.816700000
H	-2.914000000	3.200500000	0.690600000
O	-3.548400000	2.238400000	-0.618100000
H	-3.676900000	2.718200000	-1.453100000
H	-2.749500000	1.644800000	-0.776100000

RC2

E= -4311.04

Nimag=0

C	-2.107200000	2.151400000	0.498900000
Se	-1.904800000	0.175700000	0.492500000
O	0.042100000	0.546600000	1.190800000
C	-1.172300000	-0.061600000	-1.342900000
O	1.081000000	-1.990800000	0.403800000
S	2.580000000	-2.182600000	0.212600000
C	3.006900000	-1.461900000	-1.427000000
C	3.416800000	-0.887800000	1.221200000
Se	-4.345900000	-0.217800000	-0.366700000
C	-5.004300000	-0.665700000	1.469100000
S	1.839700000	2.193500000	-0.779000000
C	2.501500000	3.368700000	0.482400000
H	-5.906400000	-1.269000000	1.350000000
H	-4.242800000	-1.249600000	1.992800000
H	-5.235600000	0.246400000	2.021900000
H	-2.605700000	2.398800000	1.437400000
H	-2.731600000	2.419600000	-0.351000000
H	-1.102800000	2.569300000	0.450500000
H	-0.649900000	0.860400000	-1.597200000
H	-0.475900000	-0.896100000	-1.250500000
H	-1.991500000	-0.279700000	-2.025900000
H	0.443700000	-0.349900000	1.099900000
H	2.478200000	-2.059400000	-2.173800000
H	2.692900000	-0.413900000	-1.454800000
H	4.088600000	-1.550800000	-1.571400000
H	3.179100000	-1.105500000	2.265100000
H	4.495600000	-0.963800000	1.052300000
H	3.036200000	0.093800000	0.926100000
H	1.045200000	1.452700000	0.105600000
H	3.163900000	4.064900000	-0.037900000
H	1.687400000	3.928400000	0.949600000
H	3.069800000	2.836200000	1.249800000

PCs

DMSO+SH

PC1

E= -3012.60

Nimag=0

C	1.340500000	1.170400000	-0.470200000
S	-0.146400000	1.231400000	0.593000000
O	-1.346500000	0.783800000	-0.410300000
C	0.049400000	-0.498700000	1.268200000
H	1.375700000	2.115900000	-1.009600000
H	2.207500000	1.044500000	0.184600000
H	1.232600000	0.317100000	-1.144200000
H	0.952200000	-0.507600000	1.886100000
H	-0.832100000	-0.685000000	1.885200000
H	0.112700000	-1.228800000	0.457500000
C	0.896800000	4.211900000	1.182700000
H	1.874000000	4.411400000	0.730500000
H	0.636300000	5.046800000	1.840000000
H	0.982200000	3.307300000	1.811700000
S	-0.387300000	3.971900000	-0.112200000
H	-2.105300000	3.994600000	1.075300000
O	-3.028100000	3.785200000	1.456100000
H	-3.453800000	4.648000000	1.599500000
H	-3.384100000	2.952000000	0.015200000
O	-3.311200000	2.405500000	-0.826800000
H	-3.068300000	3.052100000	-1.513200000
H	-2.045200000	1.541800000	-0.551800000

PC2

E= -4348.26

Nimag=0

C	-2.380100000	1.776500000	0.100400000
S	-2.086200000	-0.032000000	0.139300000
O	-0.119200000	0.547900000	1.429600000
C	-1.020600000	-0.226000000	-1.341300000
O	1.353700000	-1.588600000	0.497300000
S	2.844100000	-1.423300000	0.183800000
C	2.963800000	-0.596500000	-1.454700000
C	3.448400000	0.003300000	1.172300000
S	-3.942500000	-0.787400000	-0.796800000
C	-4.742800000	-1.375300000	0.751300000
S	1.122200000	2.459900000	-0.545500000
C	1.412800000	4.121900000	0.220600000
H	-5.637600000	-1.917800000	0.431900000
H	-4.080300000	-2.057300000	1.289900000
H	-5.037500000	-0.537700000	1.387900000
H	-2.803800000	2.041400000	1.070900000
H	-3.075300000	1.993600000	-0.710800000
H	-1.397100000	2.252100000	-0.060800000
H	-0.352500000	0.641600000	-1.356100000
H	-0.462800000	-1.151200000	-1.192000000
H	-1.649200000	-0.257800000	-2.232300000
H	0.425800000	-0.257100000	1.217500000
H	2.498400000	-1.272300000	-2.177100000
H	2.461500000	0.377700000	-1.413900000
H	4.026800000	-0.475400000	-1.688800000
H	3.323700000	-0.270500000	2.222800000
H	4.509300000	0.142600000	0.939300000
H	2.860700000	0.892100000	0.908700000
H	0.358000000	1.288700000	0.882900000
H	0.665600000	4.333200000	0.993600000

H	2.407100000	4.160100000	0.678900000
H	1.354000000	4.901000000	-0.546400000

DMSO+SeH
PC1
E=-2992.22
Nimag=0

C	1.362100000	1.154100000	-0.484500000
S	-0.150200000	1.256000000	0.537800000
O	-1.323800000	0.783200000	-0.488200000
C	0.019900000	-0.453400000	1.276700000
H	1.435300000	2.094500000	-1.029400000
H	2.207400000	1.010700000	0.194700000
H	1.250800000	0.299000000	-1.155800000
H	0.903900000	-0.444300000	1.921200000
H	-0.880900000	-0.615400000	1.872300000
H	0.104000000	-1.209200000	0.491900000
C	0.992500000	4.244900000	1.229600000
H	1.989400000	4.410500000	0.814600000
H	0.730600000	5.066700000	1.898500000
H	0.986800000	3.307300000	1.806700000
Se	-0.344600000	4.123300000	-0.250000000
H	-2.143300000	4.020600000	1.063800000
O	-3.034400000	3.741400000	1.464000000
H	-3.507100000	4.571900000	1.647100000
H	-3.411600000	2.888800000	0.040400000
O	-3.369000000	2.332200000	-0.796600000
H	-3.210500000	2.975600000	-1.509800000
H	-2.055700000	1.513100000	-0.597600000

PC2
E=-4330.27
Nimag=0

C	-2.187000000	1.694300000	1.234800000
S	-2.616000000	0.288200000	0.157000000
O	0.326300000	0.038500000	1.209300000
C	-1.862700000	0.784300000	-1.380200000
O	2.526100000	-1.453300000	2.106800000
S	3.868800000	-1.044000000	1.517300000
C	3.782000000	-1.353100000	-0.296600000
C	3.885300000	0.796400000	1.401500000
Se	-5.015300000	0.731200000	0.102700000
C	-5.372300000	-0.911200000	-0.964500000
S	1.264500000	1.353000000	-1.534100000
C	0.957300000	2.997400000	-0.752000000
H	-6.456700000	-0.949300000	-1.092100000
H	-4.888200000	-0.833000000	-1.939300000
H	-5.028800000	-1.788700000	-0.414900000
H	-2.783700000	1.591300000	2.143500000
H	-2.407200000	2.632800000	0.721100000
H	-1.123000000	1.566400000	1.454500000
H	-0.716800000	0.831300000	-1.272900000
H	-2.101700000	0.022200000	-2.125800000
H	-2.223200000	1.767100000	-1.694100000
H	1.014900000	-0.562400000	1.587000000
H	3.646800000	-2.430600000	-0.418100000
H	2.944600000	-0.791200000	-0.720800000
H	4.729800000	-1.036500000	-0.744400000
H	3.802600000	1.169500000	2.425100000
H	4.841500000	1.098800000	0.962100000
H	3.047200000	1.130900000	0.784000000
H	0.720100000	0.338600000	0.345600000

H	1.055800000	2.946700000	0.338000000
H	1.670200000	3.730500000	-1.142400000
H	-0.052800000	3.351600000	-0.995800000

DMSeO+SH
PC1
E=-2996.25
Nimag=0

C	1.449800000	1.139100000	-0.489500000
Se	-0.086700000	1.269000000	0.763200000
O	-1.450500000	0.730200000	-0.290400000
C	0.110600000	-0.688400000	1.317400000
H	1.682500000	2.151200000	-0.813600000
H	2.274200000	0.653500000	0.037500000
H	1.091900000	0.525900000	-1.318800000
H	1.035500000	-0.766200000	1.896200000
H	-0.757600000	-0.919000000	1.936900000
H	0.134600000	-1.319900000	0.428000000
C	0.890300000	4.326600000	1.170200000
H	1.824400000	4.607400000	0.673900000
H	0.564300000	5.150300000	1.811600000
H	1.094600000	3.459400000	1.824800000
S	-0.408100000	3.910600000	-0.065200000
H	-2.199100000	4.050800000	1.094400000
O	-3.136900000	3.897100000	1.436400000
H	-3.524000000	4.782800000	1.543400000
H	-3.478200000	3.028500000	-0.060200000
O	-3.384500000	2.473800000	-0.884700000
H	-3.100400000	3.103300000	-1.570200000
H	-2.075100000	1.505500000	-0.493900000

PC2
E=-4335.85
Nimag=0

C	-2.404653000	1.782230000	0.214528000
Se	-2.036869000	-0.175373000	0.254881000
O	-0.044478000	0.493135000	1.312376000
C	-0.996090000	-0.329744000	-1.434146000
O	1.510719000	-1.577316000	0.468997000
S	2.999956000	-1.358254000	0.176262000
C	3.112121000	-0.551699000	-1.471307000
C	3.527585000	0.106411000	1.152877000
S	-4.042051000	-0.891532000	-0.784872000
C	-4.984229000	-1.237457000	0.761172000
S	1.095413000	2.422757000	-0.635842000
C	1.372622000	4.000343000	0.295590000
H	-5.908360000	-1.733017000	0.449965000
H	-4.423740000	-1.910652000	1.415845000
H	-5.235251000	-0.313212000	1.288199000
H	-2.666181000	2.056147000	1.237641000
H	-3.232937000	1.930691000	-0.476417000
H	-1.471165000	2.259987000	-0.112989000
H	-0.495704000	0.631975000	-1.570510000
H	-0.279778000	-1.130742000	-1.249497000
H	-1.693413000	-0.562770000	-2.237749000
H	0.545079000	-0.291151000	1.120979000
H	2.693835000	-1.258622000	-2.192446000
H	2.560816000	0.396608000	-1.450636000
H	4.172542000	-0.382084000	-1.686330000
H	3.392271000	-0.155200000	2.205139000
H	4.586378000	0.284859000	0.938823000
H	2.909642000	0.964786000	0.860802000

H	0.390580000	1.252428000	0.701793000
H	0.496252000	4.257064000	0.901479000
H	2.240052000	3.911734000	0.959212000
H	1.557561000	4.815224000	-0.411496000

DMSeO+SeH
PC1
E= -2975.67
Nimag=0

C	1.500300000	1.148500000	-0.460100000
Se	-0.102000000	1.289100000	0.704700000
O	-1.410200000	0.731300000	-0.416500000
C	0.062900000	-0.667000000	1.289700000
H	1.821700000	2.162800000	-0.687300000
H	2.253800000	0.561700000	0.069800000
H	1.153500000	0.634500000	-1.358600000
H	0.950800000	-0.739100000	1.924300000
H	-0.842900000	-0.892400000	1.854500000
H	0.141600000	-1.302800000	0.406400000
C	0.995200000	4.390500000	1.174300000
H	1.949900000	4.642500000	0.707800000
H	0.664100000	5.210800000	1.813400000
H	1.121400000	3.491600000	1.798100000
Se	-0.375700000	4.039400000	-0.236200000
H	-2.216900000	4.055900000	1.094500000
O	-3.115400000	3.824800000	1.488000000
H	-3.551500000	4.678000000	1.656300000
H	-3.524500000	2.943400000	0.019000000
O	-3.486600000	2.380500000	-0.804000000
H	-3.353400000	3.012500000	-1.531300000
H	-2.084400000	1.475200000	-0.559400000

PC2
E=-4317.77
Nimag=0

C	-2.277400000	2.271900000	0.253400000
Se	-2.170800000	0.297100000	0.422000000
O	-0.059600000	0.738700000	1.459200000
C	-1.163500000	-0.072500000	-1.263100000
O	1.191200000	-1.543600000	0.625700000
S	2.696400000	-1.533600000	0.334400000
C	2.923400000	-0.763000000	-1.318700000
C	3.419400000	-0.150900000	1.304600000
Se	-4.502500000	-0.062600000	-0.441600000
C	-4.642600000	-1.904800000	0.321700000
S	1.286400000	2.427200000	-0.592000000
C	1.474000000	4.144600000	0.076400000
H	-5.685500000	-2.201900000	0.191100000
H	-3.988300000	-2.587400000	-0.221800000
H	-4.394500000	-1.880700000	1.384000000
H	-2.570000000	2.641400000	1.236300000
H	-3.021300000	2.510100000	-0.506100000
H	-1.267700000	2.589200000	-0.037600000
H	-0.521400000	0.798800000	-1.431400000
H	-0.572900000	-0.964900000	-1.051700000
H	-1.902000000	-0.221700000	-2.048800000
H	0.415200000	-0.119000000	1.268100000
H	2.417200000	-1.413100000	-2.036900000
H	2.506000000	0.250900000	-1.314500000
H	3.998100000	-0.740200000	-1.528300000
H	3.245100000	-0.386000000	2.357500000
H	4.493600000	-0.123700000	1.094700000
H	2.930700000	0.785700000	1.009300000
H	0.448300000	1.418800000	0.828500000
H	0.541100000	4.494200000	0.534600000
H	2.266400000	4.181100000	0.831500000
H	1.736400000	4.827400000	-0.738100000

Chalcogenurane U

DMSO+SH
E=-3016.45
Nimag=0

C	0.667100000	1.511300000	-0.672000000
S	-0.212200000	1.208900000	0.910200000
O	-1.442300000	0.109300000	0.181500000
C	0.733100000	-0.216100000	1.555600000
H	0.230400000	2.420200000	-1.090700000
H	1.732200000	1.650700000	-0.486700000
H	0.464300000	0.652200000	-1.312100000
H	1.788400000	0.049100000	1.585900000
H	0.358500000	-0.405300000	2.563500000
H	0.522400000	-1.067300000	0.905900000
C	0.440000000	2.797800000	3.591200000
H	0.467500000	3.771600000	4.087700000
H	-0.608900000	2.528400000	3.408700000
H	0.894900000	2.051600000	4.249600000
S	1.349200000	2.897000000	1.991800000
H	-0.185700000	4.065800000	0.978000000
O	-0.977100000	4.396300000	0.450500000
H	-1.191000000	5.266000000	0.827900000
H	-2.344000000	3.229500000	0.227200000
O	-3.017600000	2.516400000	0.073400000
H	-3.477500000	2.771200000	-0.743700000
H	-2.197400000	0.742600000	0.100500000

DMSO+SeH
E= -2995.42
Nimag=0

C	0.698200000	1.490400000	-0.714500000
S	-0.166400000	1.210300000	0.880200000
O	-1.438500000	0.134100000	0.168000000
C	0.753900000	-0.237000000	1.514200000
H	0.303000000	2.428000000	-1.110700000
H	1.772900000	1.568500000	-0.550500000
H	0.437700000	0.652500000	-1.362400000
H	1.816500000	-0.001600000	1.526700000
H	0.391800000	-0.411800000	2.529200000
H	0.509000000	-1.084000000	0.870700000
C	0.389200000	2.805100000	3.694300000
H	0.352400000	3.776800000	4.189900000
H	-0.624900000	2.499200000	3.415100000
H	0.836500000	2.065200000	4.360900000
Se	1.490100000	2.971300000	2.033700000
H	-0.194700000	4.115000000	0.932100000
O	-0.994200000	4.405600000	0.398000000
H	-1.208000000	5.294100000	0.729600000
H	-2.362200000	3.227500000	0.257000000
O	-3.040800000	2.511000000	0.149300000
H	-3.538700000	2.748600000	-0.650500000
H	-2.188400000	0.778500000	0.123400000

DMSeO+SH

E= -3005.88
Nimag=0

C	0.487200000	1.257700000	-0.959700000
Se	-0.271100000	0.799900000	0.817400000
O	-1.678600000	-0.323900000	-0.019300000
C	0.751800000	-0.867900000	1.125400000
H	-0.063300000	2.135800000	-1.296700000
H	1.552800000	1.459200000	-0.855700000
H	0.274700000	0.396400000	-1.592100000
H	1.800000000	-0.661900000	0.916300000
H	0.611600000	-1.129400000	2.175300000
H	0.314400000	-1.618600000	0.467300000
C	0.944700000	2.119000000	3.583400000
H	1.269500000	2.998500000	4.145600000
H	-0.151500000	2.098200000	3.572700000
H	1.321500000	1.219400000	4.078500000
S	1.604100000	2.237500000	1.863200000
H	0.226100000	3.823000000	1.250300000
O	-0.545700000	4.391700000	0.955000000
H	-0.148100000	5.135900000	0.472600000
H	-1.832900000	3.424200000	0.175900000
O	-2.452100000	2.772700000	-0.245700000
H	-3.331600000	3.010100000	0.091800000
H	-2.366200000	0.347700000	-0.204000000

DMSeO+SeH
E= -2985.35
Nimag=0

C	0.521600000	1.250200000	-0.989500000
Se	-0.217200000	0.797900000	0.798900000
O	-1.691000000	-0.265300000	-0.012900000
C	0.751300000	-0.909500000	1.064100000
H	0.084300000	2.212100000	-1.257200000
H	1.608000000	1.305600000	-0.929100000
H	0.170900000	0.458400000	-1.650600000
H	1.804500000	-0.738100000	0.849500000
H	0.611400000	-1.184400000	2.110500000
H	0.281100000	-1.631100000	0.395900000
C	0.928700000	2.164300000	3.707100000
H	1.187800000	3.070700000	4.256800000
H	-0.156800000	2.110600000	3.584300000
H	1.294300000	1.285300000	4.240500000
Se	1.792900000	2.262900000	1.903200000
H	0.241300000	3.850900000	1.196400000
O	-0.558200000	4.361000000	0.876600000
H	-0.199200000	5.125500000	0.395200000
H	-1.880500000	3.369100000	0.170500000
O	-2.528400000	2.722500000	-0.210400000
H	-3.381700000	2.961500000	0.187900000
H	-2.347700000	0.445100000	-0.170300000

Transition States

DMSeO+SH
TS1
E= -3009.44
Nimag=-604

C	1.303100000	1.211200000	-0.428500000
S	-0.257700000	1.136600000	0.515300000
O	-1.359100000	0.792900000	-0.555600000
C	-0.000300000	-0.559800000	1.206900000
H	1.278100000	2.163200000	-0.960300000
H	2.125600000	1.184900000	0.292600000
H	1.338800000	0.353500000	-1.104900000
H	0.852100000	-0.532500000	1.892500000
H	-0.914100000	-0.812800000	1.749300000
H	0.167200000	-1.266900000	0.390000000
C	0.918100000	4.308400000	1.241600000
H	1.926700000	4.409300000	0.826500000
H	0.721000000	5.173700000	1.881900000
H	0.885900000	3.405100000	1.869900000
S	-0.314400000	4.208900000	-0.129600000
H	-1.854600000	4.053300000	0.881900000
O	-2.822800000	3.766000000	1.315700000
H	-3.340400000	4.584600000	1.414000000
H	-3.196900000	2.971100000	0.251800000
O	-3.300000000	2.279200000	-0.604000000
H	-3.214600000	2.826000000	-1.406400000
H	-2.337400000	1.591500000	-0.570100000

TS1-2
E= -3009.59
Nimag=-97

C	1.053300000	1.157800000	-0.942100000
S	0.047900000	1.148300000	0.573900000
O	-1.391100000	0.728400000	-0.099900000
C	0.593500000	-0.529700000	1.156500000
H	0.767100000	2.061000000	-1.480700000
H	2.099100000	1.234400000	-0.639200000
H	0.847300000	0.245700000	-1.509900000
H	1.635000000	-0.437100000	1.477800000
H	-0.045000000	-0.777000000	2.006700000
H	0.487400000	-1.263700000	0.354100000
C	0.448300000	3.841800000	2.519000000
H	1.323500000	3.919900000	3.171700000
H	-0.247200000	4.650000000	2.764600000
H	-0.056300000	2.883000000	2.726800000
S	0.965400000	3.938600000	0.751100000
H	-0.892300000	4.576500000	0.219000000
O	-1.884700000	4.815000000	0.068400000
H	-1.914100000	5.248000000	-0.801500000
H	-2.681100000	3.456500000	0.315500000
O	-3.067600000	2.540100000	0.553300000
H	-3.943300000	2.480600000	0.137800000
H	-2.089900000	1.463200000	0.166400000

TS2
E=-4336.96
Nimag=-623

C	-1.984000000	2.080600000	0.315900000
S	-1.888200000	0.244100000	0.387900000
O	0.104100000	0.578700000	1.136100000
C	-1.165900000	-0.157900000	-1.254500000
O	1.087700000	-1.958500000	0.522100000
S	2.575200000	-2.142000000	0.224100000
C	2.870600000	-1.471900000	-1.463800000
C	3.471600000	-0.809800000	1.123600000
S	-4.020700000	-0.195300000	-0.299700000
C	-4.710300000	-0.422100000	1.389800000
S	1.707000000	2.054400000	-0.755300000
C	2.367400000	3.324800000	0.417900000
H	-5.685700000	-0.901600000	1.268200000

H	-4.062100000	-1.078100000	1.978500000
H	-4.842600000	0.536200000	1.899500000
H	-2.196400000	2.409100000	1.335200000
H	-2.792400000	2.351700000	-0.361400000
H	-1.012600000	2.453500000	-0.017200000
H	-0.450400000	0.628700000	-1.497600000
H	-0.654700000	-1.112800000	-1.119500000
H	-1.962400000	-0.224100000	-1.995600000
H	0.468800000	-0.341400000	1.068100000
H	2.284200000	-2.089200000	-2.149100000
H	2.563400000	-0.420700000	-1.492900000
H	3.937800000	-1.573500000	-1.687100000
H	3.292500000	-0.978400000	2.188100000
H	4.538100000	-0.908500000	0.896800000
H	3.083600000	0.160600000	0.796600000
H	0.840000000	1.228300000	0.320200000
H	2.884700000	4.097600000	-0.157200000
H	1.553200000	3.789100000	0.982800000
H	3.075500000	2.875100000	1.121800000

DMSO+SeH
TS1
E=-2988.79
Nimag=-356

C	1.236000000	1.159400000	-0.581500000
S	-0.253000000	1.130700000	0.475900000
O	-1.420000000	0.712600000	-0.481000000
C	0.092400000	-0.487900000	1.291900000
H	1.150500000	2.066600000	-1.182200000
H	2.110700000	1.215500000	0.073500000
H	1.247200000	0.249900000	-1.188300000
H	0.999300000	-0.383500000	1.895500000
H	-0.765900000	-0.698600000	1.933800000
H	0.204900000	-1.266200000	0.532500000
C	0.984000000	4.241400000	1.380000000
H	2.016400000	4.277600000	1.023600000
H	0.810400000	5.066400000	2.073600000
H	0.805800000	3.291900000	1.899300000
Se	-0.247500000	4.412100000	-0.194700000
H	-1.933300000	4.148800000	0.844100000
O	-2.892700000	3.805100000	1.226700000
H	-3.456700000	4.594900000	1.315600000
H	-3.194000000	3.022000000	0.178300000
O	-3.261700000	2.326800000	-0.706400000
H	-3.029300000	2.870200000	-1.483500000
H	-2.400200000	1.602000000	-0.588900000

TS1-2
E= -2989.48
Nimag=-87

C	1.106600000	1.088500000	-0.977400000
S	0.095800000	1.146600000	0.534400000
O	-1.352900000	0.770600000	-0.148200000
C	0.583900000	-0.542800000	1.150300000
H	0.853000000	1.989200000	-1.536300000
H	2.153100000	1.134000000	-0.670600000
H	0.871900000	0.171100000	-1.525600000
H	1.626300000	-0.478700000	1.475600000
H	-0.066900000	-0.752400000	2.001300000
H	0.457100000	-1.287300000	0.360800000
C	0.404800000	3.839100000	2.574200000
H	1.251700000	3.900400000	3.260900000

H	-0.308800000	4.634600000	2.797200000
H	-0.093400000	2.866600000	2.692800000
Se	1.068700000	4.035600000	0.694900000
H	-0.961200000	4.688100000	0.171600000
O	-1.956000000	4.889500000	0.049100000
H	-2.023800000	5.274800000	-0.841600000
H	-2.677800000	3.483500000	0.357100000
O	-3.013900000	2.558100000	0.620800000
H	-3.922500000	2.477400000	0.287700000
H	-2.040700000	1.494800000	0.160100000

TS2
E= -4317.79
Nimag=-637

C	-1.966500000	2.183200000	0.233300000
S	-1.889400000	0.349200000	0.339200000
O	0.113900000	0.661500000	1.123200000
C	-1.151500000	-0.067000000	-1.294400000
O	1.008600000	-1.921200000	0.534900000
S	2.490800000	-2.164300000	0.256400000
C	2.834200000	-1.511900000	-1.429700000
C	3.428900000	-0.866800000	1.163700000
Se	-4.184400000	-0.110600000	-0.350600000
C	-4.718700000	-0.628500000	1.500600000
S	1.793600000	2.062400000	-0.759100000
C	2.426500000	3.366500000	0.391600000
H	-5.657600000	-1.178500000	1.407100000
H	-3.949500000	-1.278300000	1.922400000
H	-4.863000000	0.260100000	2.116500000
H	-2.266500000	2.528500000	1.224700000
H	-2.710900000	2.458100000	-0.512400000
H	-0.967200000	2.541200000	-0.023000000
H	-0.393400000	0.687500000	-1.510500000
H	-0.691200000	-1.047700000	-1.161500000
H	-1.931000000	-0.086700000	-2.055600000
H	0.448500000	-0.270300000	1.068100000
H	2.232200000	-2.107300000	-2.120700000
H	2.569700000	-0.449600000	-1.466400000
H	3.899200000	-1.656700000	-1.639400000
H	3.231200000	-1.026300000	2.226400000
H	4.493100000	-1.008500000	0.949400000
H	3.084100000	0.118100000	0.831800000
H	0.888300000	1.284200000	0.304000000
H	2.979200000	4.108300000	-0.191400000
H	1.597900000	3.864900000	0.904100000
H	3.097800000	2.933000000	1.140100000

DMSeO+SH
TS1
E= -2984.10
Nimag=-1132

C	1.462100000	1.138900000	-0.526100000
Se	-0.181800000	1.203400000	0.584300000
O	-1.411000000	0.856300000	-0.597200000
C	0.028300000	-0.720800000	1.187900000
H	1.555900000	2.124600000	-0.978500000
H	2.299300000	0.904800000	0.136600000
H	1.301300000	0.353300000	-1.266200000
H	0.910800000	-0.795200000	1.829900000
H	-0.880000000	-0.965400000	1.741400000
H	0.119900000	-1.343900000	0.296700000
C	0.871700000	4.454400000	1.391700000

H	1.898500000	4.676600000	1.086200000
H	0.490700000	5.294800000	1.979000000
H	0.881100000	3.561400000	2.034300000
S	-0.166400000	4.181900000	-0.109900000
H	-1.693900000	4.167100000	0.652500000
O	-2.767800000	3.999000000	1.122500000
H	-3.262500000	4.832600000	1.035200000
H	-3.208200000	3.106400000	0.361900000
O	-3.464100000	2.168600000	-0.328500000
H	-3.719100000	2.485300000	-1.212000000
H	-2.460700000	1.563300000	-0.451200000

TS1-2
E= -2992.24
Nimag=-86

C	1.200800000	1.164300000	-0.958200000
Se	0.165600000	1.162900000	0.723700000
O	-1.422000000	0.763700000	-0.097500000
C	0.651000000	-0.813800000	1.075600000
H	0.952800000	2.096900000	-1.462400000
H	2.257300000	1.141500000	-0.685500000
H	0.900500000	0.284400000	-1.528700000
H	1.725100000	-0.863600000	1.273500000
H	0.074500000	-1.110200000	1.953400000
H	0.372400000	-1.396600000	0.196400000
C	0.324500000	3.986100000	2.565100000
H	1.167100000	4.235200000	3.216300000
H	-0.446400000	4.754700000	2.668200000
H	-0.101600000	3.025600000	2.894200000
S	0.897500000	3.877200000	0.814100000
H	-0.941800000	4.650500000	0.121800000
O	-1.899500000	4.925900000	-0.060700000
H	-1.906400000	5.217600000	-0.988100000
H	-2.739100000	3.542200000	0.383600000
O	-3.085000000	2.660400000	0.723700000
H	-4.014400000	2.600200000	0.449000000
H	-2.077700000	1.476400000	0.211500000

TS2
E= -4329.79
Nimag=-578

C	-2.040200000	2.072400000	0.364100000
Se	-1.910400000	0.088400000	0.478700000
O	0.132000000	0.507100000	1.248200000
C	-1.087300000	-0.295200000	-1.291100000
O	1.251100000	-1.938000000	0.558100000
S	2.740100000	-2.036100000	0.223400000
C	2.955200000	-1.347900000	-1.468000000
C	3.577600000	-0.655400000	1.105700000
S	-4.114900000	-0.350100000	-0.396400000
C	-4.968400000	-0.447200000	1.234700000
S	1.597000000	2.077000000	-0.677100000
C	2.395800000	3.274400000	0.486200000
H	-5.964500000	-0.857000000	1.045100000
H	-4.434200000	-1.120200000	1.912500000
H	-5.071300000	0.542000000	1.689700000
H	-2.223700000	2.415000000	1.383700000
H	-2.875500000	2.295400000	-0.297300000
H	-1.078700000	2.432000000	-0.011300000
H	-0.526300000	0.594500000	-1.576700000
H	-0.421800000	-1.139200000	-1.107000000
H	-1.884100000	-0.533700000	-1.994000000

H	0.560000000	-0.383900000	1.149700000
H	2.393900000	-2.000100000	-2.141900000
H	2.581600000	-0.317300000	-1.485500000
H	4.021700000	-1.383100000	-1.714100000
H	3.433500000	-0.834700000	2.173800000
H	4.642400000	-0.690000000	0.853900000
H	3.123200000	0.288700000	0.788400000
H	0.789300000	1.199500000	0.437200000
H	2.870000000	4.068900000	-0.096800000
H	1.651300000	3.720300000	1.152900000
H	3.162300000	2.780000000	1.093500000

DMSeO+SeH
TS1
E=-2962.75
Nimag=-943

C	1.391000000	1.062100000	-0.694400000
Se	-0.157800000	1.181100000	0.545100000
O	-1.470800000	0.766300000	-0.504400000
C	0.141600000	-0.687600000	1.271100000
H	1.374600000	1.982100000	-1.276600000
H	2.297400000	0.987000000	-0.087500000
H	1.235800000	0.170800000	-1.304600000
H	1.093900000	-0.703700000	1.808900000
H	-0.692300000	-0.887500000	1.946300000
H	0.137200000	-1.380300000	0.427900000
C	0.913500000	4.436800000	1.581400000
H	1.967500000	4.616500000	1.359400000
H	0.522700000	5.253300000	2.191300000
H	0.808000000	3.492700000	2.128700000
Se	-0.091300000	4.344500000	-0.150700000
H	-1.781900000	4.252500000	0.628600000
O	-2.846900000	4.047100000	1.045200000
H	-3.363700000	4.866400000	0.939100000
H	-3.229800000	3.176000000	0.302900000
O	-3.473300000	2.200800000	-0.416100000
H	-3.631800000	2.498000000	-1.328700000
H	-2.539200000	1.572500000	-0.442300000

TS1-2
E= -2971.86
Nimag=-75

C	1.274700000	1.079500000	-0.990400000
Se	0.212800000	1.162500000	0.672600000
O	-1.373600000	0.826100000	-0.176900000
C	0.611100000	-0.833300000	1.073900000
H	1.062500000	2.002400000	-1.527500000
H	2.325100000	1.032800000	-0.697700000
H	0.958000000	0.190800000	-1.538300000
H	1.680100000	-0.924700000	1.283800000
H	0.013700000	-1.082900000	1.952200000
H	0.314600000	-1.422200000	0.204800000
C	0.297100000	4.007600000	2.611200000
H	1.117600000	4.232400000	3.294800000
H	-0.481200000	4.767300000	2.698900000
H	-0.128300000	3.025900000	2.855600000
Se	1.002300000	3.989600000	0.736200000
H	-1.020300000	4.760200000	0.084700000
O	-1.992000000	4.991800000	-0.053800000
H	-2.054100000	5.249700000	-0.989600000
H	-2.737800000	3.561800000	0.437300000

O	-3.027000000	2.664600000	0.786600000
H	-3.974400000	2.580800000	0.590700000
H	-2.025800000	1.518600000	0.179800000

TS2
E=-4309.70
Nimag=-600

C	-2.472200000	1.827800000	0.097300000
Se	-2.041800000	-0.112500000	0.226500000
O	-0.122000000	0.623100000	1.099700000
C	-1.086500000	-0.337200000	-1.506100000
O	1.410600000	-1.598100000	0.434600000
S	2.921200000	-1.446800000	0.254700000
C	3.190500000	-0.653200000	-1.382400000
C	3.437600000	0.001400000	1.265300000
Se	-4.250700000	-0.960500000	-0.743200000
C	-4.989700000	-1.417300000	1.058300000
S	1.167800000	2.445000000	-0.725400000
C	1.321400000	3.892500000	0.417300000
H	-5.863900000	-2.047300000	0.881300000
H	-4.243400000	-1.976000000	1.626700000
H	-5.284900000	-0.509800000	1.587000000
H	-2.728600000	2.137800000	1.111500000
H	-3.316500000	1.927000000	-0.581800000
H	-1.566700000	2.325500000	-0.258200000
H	-0.604300000	0.617700000	-1.718700000
H	-0.349100000	-1.117800000	-1.314600000
H	-1.809100000	-0.624100000	-2.268100000
H	0.443500000	-0.188000000	1.008100000
H	2.808700000	-1.351000000	-2.131900000
H	2.663000000	0.306500000	-1.409900000
H	4.268200000	-0.511800000	-1.516100000
H	3.219700000	-0.252100000	2.305500000
H	4.514200000	0.142900000	1.126000000
H	2.874400000	0.881700000	0.937800000
H	0.461400000	1.433100000	0.321900000
H	0.351000000	4.141800000	0.858900000
H	2.032900000	3.680500000	1.221800000
H	1.679800000	4.753600000	-0.153700000

Table S4: Cartesian coordinates (Å), energies (kcal mol^{-1}) and imaginary frequencies (cm^{-1}) of the optimized structures for sulfenic and selenenic acid formation. Level of theory: COSMO-ZORA-M06/TZ2P-ae // COSMO-ZORA-BLYP-D3(BJ).

DMSO+SH
RC
E= -2937.76
Nimag=0

S	-2.701259000	-0.151054000	1.055396000
C	-2.571748000	1.437504000	0.166004000
C	-1.033071000	-0.264027000	1.776711000
S	-2.352105000	-1.712285000	-0.639822000
C	-4.139124000	-1.619243000	-1.117799000
O	-1.908881000	-3.175049000	-2.179673000
H	-2.085460000	-2.701534000	-3.016066000
H	-2.764399000	-4.164852000	-2.131153000
S	-3.762788000	-5.395370000	-2.040910000
C	-2.950581000	-6.437782000	-3.346119000
H	-1.923070000	-6.677982000	-3.058490000
H	-3.516925000	-7.366942000	-3.452164000
H	-2.945128000	-5.913099000	-4.305824000
H	-4.330552000	-2.469560000	-1.771644000
H	-4.760769000	-1.700928000	-0.224404000
H	-4.342968000	-0.686152000	-1.646667000
H	-3.499224000	1.560841000	-0.395806000
H	-1.711976000	1.406374000	-0.506202000
H	-2.471839000	2.234671000	0.906113000
H	-0.286907000	-0.231498000	0.979250000
H	-0.985934000	-1.211529000	2.315438000
H	-0.907673000	0.573545000	2.465321000

TS
E= -2923.28
Nimag=-885

S	-2.701259000	-0.151054000	1.055396000
C	-2.571748000	1.437504000	0.166004000
C	-1.033071000	-0.264027000	1.776711000
S	-2.352105000	-1.712285000	-0.639822000
C	-4.139124000	-1.619243000	-1.117799000
O	-1.908881000	-3.175049000	-2.179673000
H	-2.085460000	-2.701534000	-3.016066000
H	-2.764399000	-4.164852000	-2.131153000
S	-3.762788000	-5.395370000	-2.040910000
C	-2.950581000	-6.437782000	-3.346119000
H	-1.923070000	-6.677982000	-3.058490000
H	-3.516925000	-7.366942000	-3.452164000
H	-2.945128000	-5.913099000	-4.305824000
H	-4.330552000	-2.469560000	-1.771644000
H	-4.760769000	-1.700928000	-0.224404000
H	-4.342968000	-0.686152000	-1.646667000
H	-3.499224000	1.560841000	-0.395806000
H	-1.711976000	1.406374000	-0.506202000
H	-2.471839000	2.234671000	0.906113000
H	-0.286907000	-0.231498000	0.979250000
H	-0.985934000	-1.211529000	2.315438000
H	-0.907673000	0.573545000	2.465321000

Sulfenic
E= -966.87

Nimag=0

C	0.486200000	2.966200000	3.755300000
H	0.722400000	3.888000000	4.302400000
H	-0.598100000	2.891100000	3.626500000
H	0.872500000	2.107300000	4.311900000
S	1.345100000	3.127100000	2.153900000
O	1.028000000	1.597500000	1.459600000
H	0.218000000	1.695600000	0.924400000

DMSO+SeH
RC
E= -2923.26
Nimag=0

S	-2.661200000	-0.105700000	1.033700000
C	-2.546800000	1.523600000	0.216000000
C	-1.052700000	-0.163300000	1.891800000
Se	-2.224100000	-1.637600000	-0.689400000
C	-4.169200000	-1.750300000	-1.137000000
O	-1.693300000	-3.300800000	-2.517400000
H	-1.881400000	-2.825800000	-3.349200000
H	-2.430000000	-4.024100000	-2.449300000
S	-3.869400000	-5.426600000	-2.234600000
C	-3.259700000	-6.634800000	-3.519700000
H	-2.253100000	-6.985700000	-3.269900000
H	-3.928300000	-7.500500000	-3.567900000
H	-3.230000000	-6.161900000	-4.506700000
H	-4.263700000	-2.629600000	-1.775400000
H	-4.726400000	-1.891600000	-0.211200000
H	-4.473600000	-0.845900000	-1.662300000
H	-3.415100000	1.605200000	-0.439300000
H	-1.617300000	1.582900000	-0.352000000
H	-2.589200000	2.282300000	1.001200000
H	-0.243500000	-0.029700000	1.171700000
H	-0.992300000	-1.134800000	2.383000000
H	-1.060000000	0.642000000	2.628900000

TS
E= -2916.32
Nimag=-884

S	-2.666500000	-0.102700000	1.118200000
C	-2.566800000	1.489500000	0.226700000
C	-1.005200000	-0.165100000	1.871400000
Se	-2.262100000	-1.772200000	-0.691800000
C	-4.203800000	-1.648900000	-1.162300000
O	-1.884600000	-3.259000000	-2.290700000
H	-2.052900000	-2.789700000	-3.131200000
H	-2.728200000	-4.202800000	-2.228500000
S	-3.767000000	-5.439300000	-2.116400000
C	-2.970200000	-6.520100000	-3.401300000
H	-1.947900000	-6.774000000	-3.106700000
H	-3.552000000	-7.441200000	-3.494800000
H	-2.951400000	-6.012300000	-4.370000000
H	-4.408900000	-2.506500000	-1.802400000
H	-4.787100000	-1.714300000	-0.243800000
H	-4.392700000	-0.714300000	-1.690200000
H	-3.484100000	1.579500000	-0.358000000
H	-1.692600000	1.488200000	-0.427300000
H	-2.510900000	2.293400000	0.964300000
H	-0.242400000	-0.105300000	1.091600000
H	-0.937400000	-1.113200000	2.407200000
H	-0.916900000	0.671700000	2.567000000

Selenenic
E= -949.62
Nimag=0

C	0.469200000	2.994400000	3.821500000
H	0.715100000	3.895400000	4.394500000
H	-0.609200000	2.930200000	3.666500000
H	0.855000000	2.109600000	4.331000000
Se	1.406700000	3.196800000	2.084400000
O	1.027200000	1.515200000	1.377900000
H	0.210100000	1.631200000	0.858300000

DMSeO+SH
RC
E= -2922.68
Nimag=0

Se	-2.764300000	-0.081400000	1.144000000
C	-2.596200000	1.671000000	0.235500000
C	-0.951800000	-0.136500000	1.925000000
S	-2.334000000	-1.500100000	-0.567300000
C	-4.082600000	-1.810300000	-1.058000000
O	-1.380500000	-3.345500000	-2.762700000
H	-1.676700000	-2.759900000	-3.482900000
H	-2.157300000	-3.981600000	-2.618300000
S	-3.759700000	-5.317900000	-2.244800000
C	-3.208800000	-6.709000000	-3.361800000
H	-2.326500000	-7.208200000	-2.947500000
H	-4.008400000	-7.449500000	-3.468700000
H	-2.956100000	-6.324500000	-4.355500000
H	-4.019400000	-2.615800000	-1.795900000
H	-4.661400000	-2.151800000	-0.198500000
H	-4.519100000	-0.918200000	-1.508600000
H	-3.466300000	1.760000000	-0.414400000
H	-1.661600000	1.672700000	-0.323800000
H	-2.610200000	2.422400000	1.027100000
H	-0.228000000	-0.007700000	1.120900000
H	-0.859700000	-1.103700000	2.416500000
H	-0.917300000	0.686300000	2.639600000

TS
E= -2904.88
Nimag=-761

Se	-2.801800000	-0.133100000	1.183100000
C	-2.619800000	1.543500000	0.143600000
C	-0.949300000	-0.206700000	1.864200000
S	-2.377700000	-1.761800000	-0.582300000
C	-4.147800000	-1.687600000	-1.126400000
O	-1.852200000	-3.264300000	-2.141400000
H	-1.972700000	-2.760500000	-2.969600000
H	-2.789900000	-4.248700000	-2.170900000
S	-3.830100000	-5.402200000	-2.174300000
C	-2.973200000	-6.474000000	-3.424200000
H	-1.990400000	-6.780600000	-3.055500000
H	-3.584800000	-7.364400000	-3.592200000
H	-2.856300000	-5.938200000	-4.370400000
H	-4.269900000	-2.469600000	-1.875700000
H	-4.807600000	-1.888200000	-0.280300000
H	-4.368600000	-0.713200000	-1.566500000
H	-3.551200000	1.666000000	-0.409800000
H	-1.767100000	1.434300000	-0.526700000
H	-2.479000000	2.357000000	0.856700000
H	-0.268400000	-0.188000000	1.012400000
H	-0.864200000	-1.137300000	2.425300000

H	-0.809400000	0.659200000	2.511600000

DMSeO+SeH

RC

E= -2907.09

Nimag=0

Se	-2.729200000	-0.056000000	1.165200000
C	-2.552000000	1.682700000	0.228500000
C	-0.920500000	-0.094500000	1.964800000
Se	-2.244100000	-1.622800000	-0.662600000
C	-4.181400000	-1.805100000	-1.126300000
O	-1.622300000	-3.306900000	-2.581900000
H	-1.834400000	-2.814000000	-3.396800000
H	-2.356600000	-4.022900000	-2.509100000
S	-3.821000000	-5.436500000	-2.276100000
C	-3.155200000	-6.720600000	-3.456000000
H	-2.187600000	-7.096700000	-3.108100000
H	-3.850800000	-7.563100000	-3.527600000
H	-3.024100000	-6.291600000	-4.454900000
H	-4.223800000	-2.643000000	-1.823900000
H	-4.738700000	-2.039500000	-0.219700000
H	-4.530900000	-0.885500000	-1.593900000
H	-3.421900000	1.766700000	-0.422700000
H	-1.621300000	1.671100000	-0.337500000
H	-2.557500000	2.453900000	1.000500000
H	-0.185700000	0.019500000	1.168300000
H	-0.829300000	-1.054400000	2.471500000
H	-0.884500000	0.736300000	2.669900000

TS

E= -2897.11

Nimag=-886

Se	-2.764500000	-0.075200000	1.234300000
C	-2.584400000	1.601700000	0.190800000
C	-0.920400000	-0.120800000	1.951900000
Se	-2.299600000	-1.816200000	-0.642600000
C	-4.233900000	-1.716600000	-1.155700000
O	-1.863500000	-3.326300000	-2.248400000
H	-1.978300000	-2.834700000	-3.084900000
H	-2.769100000	-4.265300000	-2.252900000
S	-3.838500000	-5.440300000	-2.230500000
C	-2.986400000	-6.544000000	-3.457600000
H	-2.003600000	-6.844800000	-3.083700000
H	-3.599000000	-7.437200000	-3.606900000
H	-2.867900000	-6.030000000	-4.415800000
H	-4.379700000	-2.498000000	-1.901100000
H	-4.845000000	-1.913800000	-0.275100000
H	-4.446900000	-0.734600000	-1.577900000
H	-3.503500000	1.703100000	-0.387200000
H	-1.715100000	1.507400000	-0.460100000
H	-2.476700000	2.423600000	0.900100000
H	-0.219800000	-0.093500000	1.116700000
H	-0.832400000	-1.049700000	2.515600000
H	-0.803400000	0.746600000	2.601900000
